# Metabolic dysfunction-associated steatotic liver disease accelerates pancreatic cancer progression and metastasis via the macrophage migration inhibitory factor-CD44 axis

**DOI:** 10.1038/s41392-025-02562-8

**Published:** 2026-01-16

**Authors:** Qian Yu, Hui Song, Xiao-ya Shi, Liang Zhu, Yu Liang, Rui-ning Gong, Xiao-wu Dong, Shang-long Liu, Hai-zhen Wang, Ying-luo Wang, Jiu-fa Cui, Xiao-nan Yang, Ying Chen, Chao Gao, Zhan Yang, Qing-tian Zhu, Chang Li, Huan Zhang, Jie-er Ying, Mei-fang Zheng, Yan-tao Tian, Hai-tao Hu, Xin-xin Shao, Yue Li, Ming-guang Mo, Yun Lu, Zheng Ma, Shun-li Fu, Qing-hui Niu, Yuan-yu Liao, Chen-yang Zhao, Xin Liu, Ashok K. Saluja, Ji-gang Wang, Xiao-yu Li, Song-yue Guo, Wei-hua Wang, Song Wang, Bin Liu, Guo-tao Lu, He Ren

**Affiliations:** 1 Tumor Immunology and Cytotherapy of Medical Research Center, Shandong Provincial Key Laboratory of Clinical Research for Pancreatic Diseases, the Affiliated Hospital of Qingdao University, Qingdao University, Qingdao, China; 2 Department of Radiology, Peking Union Medical College Hospital, Chinese Academy of Medical Science, Beijing, China; 3 Gastrointestinal Cancer Institute (Pancreatic Disease Institute), the Affiliated Hospital of Qingdao University, Qingdao, China; 4 Pancreatic Center, Department of Gastroenterology, Yangzhou Key Laboratory of Pancreatic Disease, the Affiliated Hospital of Yangzhou University, Yangzhou University, Yangzhou, China; 5 Department of Gastrointestinal Surgery, the Affiliated Hospital of Qingdao University, Qingdao, China; 6 Department of Endocrinology and Metabolism, the Affiliated Hospital of Qingdao University, Qingdao, China; 7 Department of Radiology, the Affiliated Hospital of Qingdao University, Qingdao, China; 8 School of Biomedical Sciences, the Chinese University of Hong Kong, Hong Kong, China; 9 Department of Food Science and Nutrition and Research Center for Chinese Medicine Innovation, the Hong Kong Polytechnic University, Hong Kong, China; 10 Department of Pancreatic and Gastric Surgery, Zhejiang Cancer Hospital, Hangzhou, China; 11 Department of Pancreatic and Gastric Surgery, National Cancer Center/National Clinical Research Center for Cancer/Cancer Hospital, Chinese Academy of Medical Sciences and Peking Union Medical College, Beijing, China; 12 Department of Medicinal Chemistry, Immunophage Biotech Co. Ltd, Nanjing, China; 13 Department of Medicinal Chemistry, Cobio Biotechnology Co. Ltd, Shanghai, China; 14 Department of Liver Diseases, the Affiliated Hospital of Qingdao University, Qingdao, China; 15 Department of Gastroenterology, Zigong Fourth People’s Hospital, Zigong, China; 16 Pancreas Center, Center for Interventional Endoscopy, AdventHealth Orland, Orlando, FL USA; 17 Department of Pathology, the Affiliated Hospital of Qingdao University, Qingdao, China; 18 Department of Gastroenterology, the Affiliated Hospital of Qingdao University, Qingdao, China

**Keywords:** Tumour immunology, Gastrointestinal cancer, Metastasis

## Abstract

Pancreatic ductal adenocarcinoma (PDAC) is a highly aggressive malignancy with a poor prognosis, particularly in the presence of liver metastases. The mechanisms by which metabolic dysfunction-associated steatotic liver disease (MASLD), formerly known as nonalcoholic fatty liver disease (NAFLD), influences PDAC progression and metastasis remain poorly understood. This study investigates the role of MASLD in fostering an immunosuppressive microenvironment conducive to PDAC liver metastases and identifies the macrophage migration inhibitory factor (MIF)-CD44 axis as a key mediator of this process. Utilizing data from the UK Biobank (450,754 participants, median follow-up 14.5 years), we observed an overall increased risk of PDAC in the MASLD population (HR: 3.48; 95% CI: 2.69–4.50; P < 0.0001). Clinical cohorts confirmed the strong association between MASLD and hepatic metastases (OR: 7.06; 95% CI: 4.62–10.78; P < 0.0001). Experimental mouse models demonstrated that MASLD enhances tumor cell stemness, immune evasion, and focal adhesion in metastatic liver tissues. Mechanistically, MASLD-induced MIF secretion promotes CD44-positive PDAC cell migration, stemness, and adhesion. Targeting MIF, either genetically or pharmacologically using the MIF tautomerase inhibitor IPG1576 significantly attenuated liver metastasis in preclinical models. Validation in patient samples revealed elevated hepatic MIF and CD44 expression in MASLD-associated PDAC liver metastases. This study highlights the MIF-CD44 axis as a promising therapeutic target and underscores the importance of tailoring treatments for PDAC patients with concurrent MASLD.

## Introduction

Pancreatic ductal adenocarcinoma (PDAC) is one of the deadliest malignancies, characterized by late-stage diagnosis, high metastatic potential, and limited treatment options.^1^ Despite advancements in localized PDAC therapies, the five-year survival rate for metastatic cases remains a dismal 3%.^2^ Liver metastasis is the key determinant of poor prognosis, yet the influence of the hepatic microenvironment on PDAC progression is not fully elucidated. Metabolically-dysfunction-associated steatotic liver disease (MASLD), previously referred to as nonalcoholic fatty liver disease (NAFLD), has emerged as a significant comorbidity associated with increased cancer risk, including gastrointestinal malignancies. MASLD prevalence has risen substantially, from 25.3% to 38.2% over the past three decades in the United States, paralleling the global obesity epidemic.^3^ Approximately 50% of patients undergoing pancreatic cancer surgery develop MASLD within 12 months postoperatively.^4^ Retrospective studies indicate that MASLD contributes to higher rates of liver metastasis and recurrence following cancer resection.^5–7^ Consequently, the rising rates of MASLD are likely to contribute to an increase in liver metastasis incidence, as seen in colorectal cancer.^5,6,8,9^ The association between MASLD and pancreatic cancer is less clear, with studies showing inconsistent results.^10–14^ A recent large-scale prospective study involving one million individuals found an association between MASLD and an increased risk of pancreatic cancer, but it relied on the fatty liver index for diagnosis, which is less accurate than computed tomography (CT), ultrasound, or magnetic resonance imaging (MRI).^14^ Given the prevalence of hepatic metastasis in various cancers and the presence of steatosis, the lack of information on the relationship between MASLD and PDAC liver metastasis is surprising. Meanwhile, the specific mechanisms by which MASLD exacerbates PDAC metastasis remain unclear.

This study aims to reveal the correlation between MASLD and liver metastasis progression using multiple clinical cohorts. Due to the limited occurrence of surgical resections in PDAC patients with liver metastasis, this study primarily employed animal models for in vivo simulation. The murine liver metastasis model, which closely aligns with clinical pathological features, was used.^15^ This study also aims to identify the driving forces and key molecular features of this process, as treatment approaches for liver metastases may vary between individuals with or without fatty liver. We performed a comprehensive bioinformatics analysis to characterize cell–cell communication changes in metastatic liver tissues and screened for ligand-receptor pairs with enhanced interaction between MASLD liver and tumors: hepatocyte macrophage migration inhibitory factor (MIF) and pancreatic cancer cell surface receptor CD44. Emerging evidence suggests that MASLD induces a pro-tumorigenic microenvironment characterized by immunosuppression, increased fibrosis, and alterations in tumor cell behavior.^16^ These changes may be mediated by hepatic secreted factors, such as the MIF, a multifunctional cytokine with established roles in inflammation and cancer progression. MIF has been implicated in metastasis via interactions with the CD44 receptor in laryngeal cancer,^17^ a marker of cancer stem cells that enhances tumor cell adhesion and migration. In recent years, based on the establishment of hepatocyte-specific *Mif* knockout mouse models, studies have shown that hepatocytes are the primary source of MIF in the progression of MASLD and alcoholic liver disease (ALD).^18,19^ In the progression of MASLD, MIF secreted by liver cells polarizes natural killer T cells towards the pro-inflammatory and pro-fibrotic type I phenotype^18^; in ALD, MIF is an important danger signal released by liver cells in response to ethanol-induced injury.^19^ Furthermore, recent studies have emphasized the pro-tumorigenic role of MIF in several cancer types.^20–23^ In pancreatic cancer, exosomal MIF produced by pancreatic cancer cells has been shown to have a role in liver metastasis. This is supported by the observation that exosomes lacking MIF are unable to facilitate the development of the pre-metastasis niche.^23^ More recently, a highly potent small-molecule inhibitor targeting the tautomerase activity of MIF, known as IPG1576, was developed, which substantially suppressed tumor growth in an orthotopic pancreatic cancer model.^24^ Given the multiple roles of MIF in both MASLD and PDAC, we hypothesize that MIF may be a key factor mediating fatty liver metastasis and a promising therapeutic target.

In this study, we integrate epidemiological, clinical, and preclinical evidence to investigate the role of MASLD in PDAC liver metastasis. We explore the mechanistic basis of MIF-CD44 interactions and assess the therapeutic potential of targeting this axis in preclinical models. By elucidating the impact of MASLD on PDAC progression, we aim to identify novel therapeutic strategies for this high-risk patient population.

## Results

### MASLD correlates with PDAC and accelerates liver metastasis

**Fig. 1:**
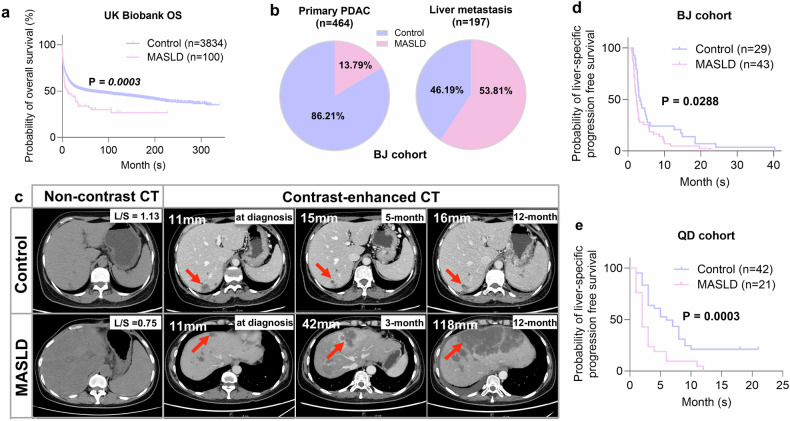
MASLD correlates with PDAC and accelerates liver metastasis. **a** Kaplan-Meier estimates of MASLD in Control (n = 3834) and MASLD (n = 100) populations. **b** The proportion of MASLD in patients with primary PDAC or liver metastasis in BJ cohort. **c** Representative non-contrast and contrast-enhanced CT from metastatic PDAC patients with or without MASLD in QD cohort. Hepatic metastases were indicated by the red arrows. **d**, **e** Probability of liver-specific progression-free survival in QD cohort and BJ cohort

Using prospective and retrospective cohorts, we analyzed associations between MASLD and PDAC/liver metastasis. After exclusions, 450,754 participants (208,907 males, 241,847 females; median age 57 [inter-quartile range: 50–63 years]) were included (Supplementary Fig. 1a). A total of 6729 (1.49%) participants were diagnosed with severe MASLD either at baseline or during the follow-up period (438 cases at baseline; 6291 cases during the follow-up) (Supplementary Fig. 1a). Baseline data are detailed in Supplementary Table 1. The incidence pattern of PDAC among individuals with MASLD is presented in Supplementary Table 2. Over a median follow-up period of 14.5 years (interquartile range: 13.76–15.22 years), 2804 participants (0.6%) were diagnosed with PDAC. Among these, 126 cases were identified in individuals with severe MASLD, while 2678 cases were found in those without MASLD. MASLD patients had a fourfold higher PDAC incidence (173 vs. 43/100,000 person-years) and significantly elevated cumulative risk (Supplementary Fig. 1b). Cox analysis showed MASLD increased PDAC risk (adjusted HR 3.48, 95% CI 2.69–4.50; P < 0.0001) (Table 1). MASLD patients also had lower post-PDAC survival (P = 0.0003; Fig. 1a). Notably, we adjusted for diabetes history within our multivariable Cox regression (Model 3) and MASLD remained independently associated with PDAC risk (HR = 3.48, 95% CI 2.69–4.50, P < 0.0001), suggesting its contribution to PDAC extends beyond diabetic comorbidity (Table 1).

**Table 1:** Associations between MASLD as time-varying exposures and subsequent PDAC as the outcome

	Hazard ratio (95% CI)	P value
	MASLD exposure (time varying)	
MASLD		
Model 1^a^	4.28 (3.31, 5.53)	<0.0001
Model 2^b^	3.73 (2.88, 4.82)	<0.0001
Model 3^c^	3.48 (2.69, 4.50)	<0.0001

*CI* confidence interval, *TG* triglycerides

^a^Model 1, adjusted for sex, age, and Townsend deprivation index

^b^Model 2, included covariates from model 1 plus drinking status, smoking status, body mass index

^c^Model 3, included covariates from model 2 plus TG, history of hypertension and history of diabetes

We next utilized retrospective cohorts to examine the associations between MASLD and PDAC liver metastasis. From 2013 to 2023, 1127 patients were diagnosed with PDAC at Peking Union Medical College Hospital (BJ cohort), with 661 having measurable liver and spleen density. The Zhejiang Cancer Hospital (ZJ cohort) included 276 patients from 2020 to 2023, and the Affiliated Hospital of Qingdao University (QD cohort) enrolled 63 patients from 2016 to 2020. Detailed baseline characteristics of these cohorts are provided in Supplementary Table 3. In the BJ cohort, the liver metastasis rate in patients with concomitant MASLD was more than three times higher than in those without MASLD (59.41%, 106/170 vs. 16.50%, 91/491, P < 0.0001). In contrast, no statistically significant differences were observed between patients with MASLD and the non-MASLD control group regarding the rates of lung metastasis (17.06% vs. 11.61%, P = 0.0687) or peritoneal metastasis (15.29% vs. 12.83%, P = 0.4174) (Supplementary Table 3).

Interestingly, PDAC patients with MASLD were diagnosed at a significantly younger age compared to those without fatty liver in both the BJ cohort (P = 0.0006) and the ZJ cohort (P = 0.0010). A multivariate analysis of prognostic factors for hepatic metastasis in the BJ cohort was presented in Supplementary Table 4. Based on the liver and spleen density ratio from noncontrast CT, approximately 25.7% of all PDAC patients had fatty liver, which was consistent with the global MASLD prevalence. However, this rate significantly increased to 53.81% in patients with hepatic metastasis (vs. Primary PDAC, 13.79%) (Supplementary Table 4, Fig. 1b). In the BJ cohort, the risk of PDAC liver metastases was significantly higher in MASLD patients compared to controls (OR 7.06, 95% CI 4.62–10.78, P < 0.0001) (Supplementary Table 5). Similar findings were observed in the ZJ cohort (OR 3.30, 95% CI 1.32–8.24, P = 0.0106) (Supplementary Table 6). These analyses suggest a strong correlation between MASLD and hepatic metastasis. Further data from patients undergoing multiple MRI sessions revealed a marked increase in liver metastasis progression in those with combined fatty liver in both BJ cohort (P = 0.0288) and QD cohort (P = 0.0003) (Fig. 1c-e). These findings indicate MASLD correlates strongly with PDAC development, liver metastasis, and poorer survival.

Given that type 2 diabetes (T2D) is a well-established risk factor for PDAC development,^25^ we also analyzed the dual comorbidity of T2D and MASLD within our cohorts. Notably, within the BJ cohort, patients with dual comorbidities (T2D/MASLD) exhibited a significantly higher risk of liver metastasis at diagnosis compared to those with MASLD alone (OR 10.40, 95% CI 4.81–22.72; vs OR 8.10, 95% CI 4.82–13.64) (Supplementary Table 4). Furthermore, overall survival appeared shorter in patients with dual comorbidities than in those with MASLD alone (Supplementary Fig. 1c).

### MASLD fosters a supportive microenvironment for PDAC hepatic metastasis

**Fig. 2:**
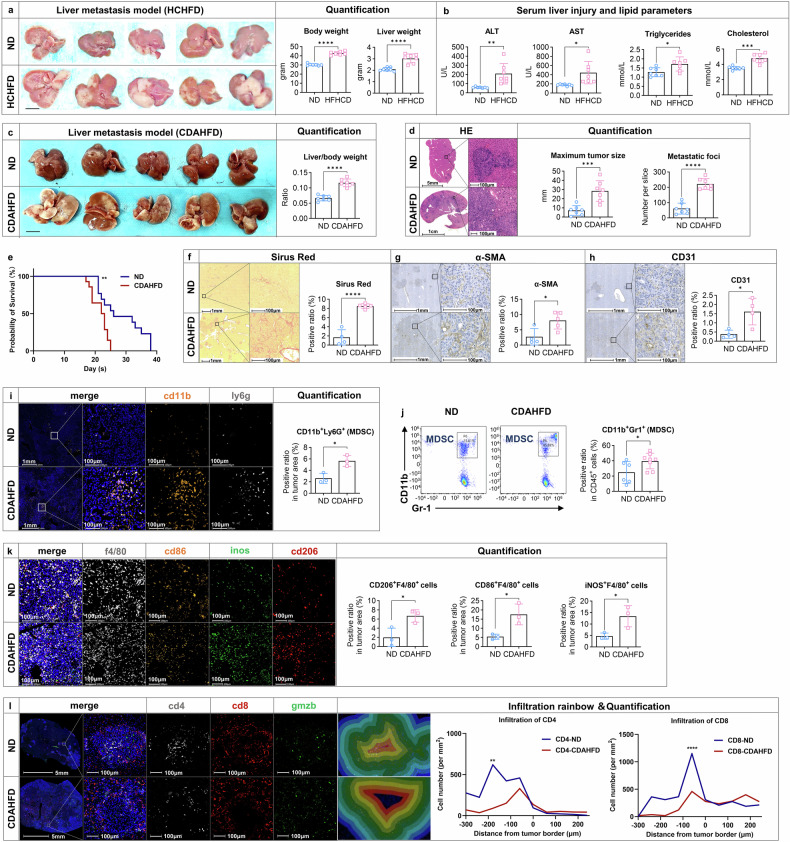
MASLD fosters a supportive microenvironment for PDAC hepatic metastasis. After 31 weeks of HCHFD feeding, mice were injected with 10^6^ KPC cells in the spleen and sacrificed on day 15 (n = 7) (**a**, **b**). **a** Representative macroscopic appearance of the liver metastases, body weight and liver weight at sacrifice. **b** Levels of serum liver injury markers, ALT and AST, triglycerides and cholesterol. After 4 weeks of CDAHFD feeding, mice were injected with 10^6^ KPC cells in the spleen and sacrificed on day 15 (n = 7). **c** Representative macroscopic appearance of the liver metastases and the liver to body weight ratio in the ND- and CDAHFD-fed groups. **d** HE staining of liver metastases with quantification of maximum tumor size and the number of metastatic foci in the ND- and CDAHFD-fed groups. **e** Cumulative survival curves in PDAC liver metastasis mouse model stratified in the ND- and CDAHFD-fed groups (n = 9 in ND group and n = 23 in CDAHFD group). **f–h** Fibrosis was assessed by Sirius Red staining and IHC staining of α-SMA, and angiogenesis was assessed by IHC staining of CD31 with quantification in CDAHFD liver metastasis model using Image J (n = 4–5). **i** The expression of CD11b and Ly6G were examined mIHC, and the ratio of CD11b^+^Ly6G^+^ cells in the tumor area was quantified by Halo software using HighPlex FL v4.2.14 module (n = 3). **j** CD45^+^ immune cells were enriched from single cell suspension of liver metastases in ND- and CDAHFD-fed mice. CD11b^+^Gr-1^+^ cells were analyzed by flow cytometry (n = 6–8/group). **k** The expressions of CD86, iNOS, CD206 and F4/80 were examined mIHC, and the ratio of CD86^+^F4/80^+^, iNOS^+^F4/80^+^ and CD206^+^F4/80^+^ cells in the tumor area was quantified by Halo software using HighPlex FL v4.2.14 module (n = 3), respectively. **l** Immuno-suppressive microenvironment of liver metastasis was examined by mIHC staining of CD4, CD8 and GMZB. Infiltration rainbow was shown with a band of 60μm per area and the infiltrated cell number of CD4 and CD8 per mm^2^ tissue across the tumor border was quantified (n = 3–5). Scale bar was indicated in the individual figures. Representative pictures are shown. Data are shown as mean ± SD per group. Unpaired parametric Student’s *t* test was performed to identify differences between two groups. For proximity analyses, two-way ANOVA followed by Šídák’s multiple comparison test was performed to identify differences between two groups at different distances. A *p*-value less than 0.05 was considered statistically significant. *p < 0.05, ** *p* < 0.01, *** *p* < 0.001, and **** *p* < 0.0001; “n.s.” indicates not significant. ALT, alanine aminotransferase; AST, aspartate transaminase; ND normal diet; CDAHFD choline-deficient, L-amino acid-defined, high-fat diet

To investigate the causality between MASLD and hepatic metastasis in PDAC mice, we employed three established diet-induced models: high-fat high-cholesterol diet (HFHCD), methionine-choline-deficient diet (MCD), and choline-deficient, L-amino acid-defined high-fat diet (CDAHFD).^26,27^ The HFHCD model recapitulate key features of metabolic dysfunction, while the MCD model mimics human MASH histology whereas it lacks metabolic abnormalities like weight gain.^28^ Similarly, CDAHFD is another commonly used model which closely replicates the pathological characteristics of clinical MASLD.^29^ In the liver metastasis model, all diet-induced models demonstrated a consistent enhancement in hepatic metastasis burden as shown by necropsy and histology (Fig. 2a-d and Supplementary Fig. 2a-b). Specifically, HFHCD-fed mice exhibited increased weight gain, elevated liver injury markers (ALT, AST), and higher serum triglycerides and cholesterol levels (Fig. 2a, b). CDAHFD-fed mice exhibited shorter survival, larger metastases, greater hepatic lesion coverage, and increased liver weights versus ND-fed controls (Fig. 2c-e). Oil Red O staining confirmed MASLD establishment pre-metastasis (Fig. S2c), aligning with prior findings.^30^ In orthotopic PDAC model, the incidence of hepatic metastasis was significantly increased in the CDAHFD group relative to the ND group (70.6% vs. 33.3%; data combined from batch-1 [Supplementary Fig. 2d] and batch-2 [Supplementary Fig. 5j]) (Supplementary Fig. 2e).

We next examined how CDAHFD-induced MASLD affects the tumor microenvironment in PDAC liver metastases. This model promoted fibrotic stroma consisting of activated cancer-associated fibroblasts (CAFs) as assessed by α-SMA, Sirius Red staining and increased angiogenesis as evident by increased CD31 expression (Fig. 2f-h). Since the dynamic interactions between CAFs and immune cells orchestrate processes such as metastasis and contribute to immunosuppression in PDAC,^31–33^ we next analyzed myeloid-derived immune cells (MDSCs, TAMs, DCs) and lymphoid immune cells (CD4⁺ T cells, CD8⁺ T cells, Tregs, NK cells) within the MASLD metastatic microenvironment by flow cytometry and/or multiplex immunofluorescence (mIHC) staining. Hepatic metastatic lesions under fatty liver conditions exhibited a significant increase in MDSC infiltration (Fig. 2i-j) and a pronounced enrichment of both M1- and M2-polarized TAMs (Fig. 2k), while DC populations remained unchanged (Supplementary Fig. 2f). In addition, while the proportion of total CD4 memory-activated T cells did not significantly differ between the two metastatic foci groups, their spatial localization exhibited distinct patterns (Fig. 2l and Supplementary Fig. 2g). In the control group, CD4^+^ T cells were concentrated within the tumor center, whereas in the fatty liver group, they were marginalized toward the tumor edge (Fig. 2l). Similarly, CD8^+^ T cells were even slightly more abundant in the MASLD group as shown by both flow cytometry and mIHC, yet their primary distribution remained at the tumor edge, and their cytotoxic function was unaltered (Fig. 2l and Supplementary Fig. 2h-j). The exclusion of CD4 and CD8 T cells at the tumor periphery might suggest reduced immune surveillance and anti-tumor responses, and increased immunosuppression in the context of fatty liver. Treg and NK cell populations remained unchanged (Supplementary Fig. 2k-m). Thus, the CDAHFD-driven microenvironment is associated with increased MDSC and M2-TAM accumulation alongside altered T cell distribution in metastases. Taken together, fatty liver promotes PDAC liver metastasis progression in vivo and established a metastatic microenvironment characterized by increased fibrosis, enhanced angiogenesis, and immunosuppression.

### Liver metastasis progression involves the MIF-CD44 axis triggered by MASLD

**Fig. 3:**
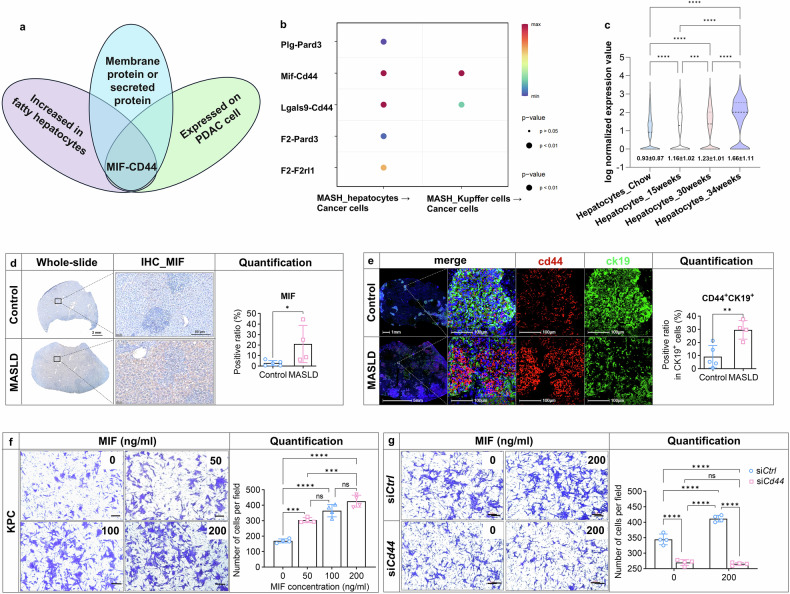
Liver metastasis progression involves the MIF-CD44 axis triggered by MASLD. **a** Venn diagram showing screening of key molecules that mediate MASLD-induced metastasis using single cell RNA-seq database GSE166504 and GSE125588, with the criteria of membrane protein or secreted protein. **b** A total of five ligand-receptor combinations were within the screening criteria. The ligands were produced from hepatocytes or Kupffer cells during NASH and the receptors were from pancreatic cancer cells of KIC mice. **c** Quantification of *Mif* RNA expression in the hepatocytes with different duration of high fat feeding based on GSE166504. **d** Representative figures and quantification of IHC of MIF expression in the liver metastasis tissues with or without MASLD (n = 4–5/group). **e** The expressions of CD44 and CK19 were examined by mIHC, and the ratio of CD44^+^ cancer cells was quantified by Halo software using HighPlex FL v4.2.14 module (n = 4/group). **f** MIF protein with different concentrations (0, 50, 100, 200 ng/ml) was added in the lower chambers of 24-well transwell plates and 5 × 10^4^ KPC cells were seeded in the upper chambers, along with 50 μg/mL (SDF-1) serving as a positive control. After 36 h of incubation, we performed staining of the invaded cells on the lower membrane surface with 0.1% crystal violet and counted their numbers. Data was collected from four independent replicate experiments. **g** KPC cells were transfected with si*Ctrl* or si*Cd44* for 24 h. MIF at a concentration of 200 ng/ml was added in the lower chambers of 24-well transwell plates with 5 × 10^4^ KPC cells in the upper chambers. After incubation for 36 h, the invaded cells crossing the membrane into lower chamber were stained with 0.1% crystal violet, and the number of migratory cells was counted. Scale bar was indicated in the individual figures. Data were collected from four independent replicate experiments. Representative pictures are shown. Data are shown as mean ± SD per group. Unpaired parametric Student’s *t*-test or one-way ANOVA was performed to identify differences between two groups or four groups, respectively. A *p*-value less than 0.05 was considered statistically significant. *p < 0.05, ** *p* < 0.01, *** *p* < 0.001, and **** *p* < 0.0001; “n.s.” indicates not significant

Hepatocytes and Kupffer cells which are the main functional cells and constitute approximately 75% of the total cells in the liver.^34^ Early interactions between these cells and tumor cells may affect the ability of extravasated disseminated tumor cells (DTCs) to establish metastases.^16^ To elucidate the underlying mechanisms through which fatty liver attracts PDAC tumor cells in vivo, we first integrated two single-cell RNA-sequencing (scRNA-Seq) datasets, GSE166504 which analyzes changes in individual liver cells across different stages of fatty liver, and GSE125588 to identify ductal cells isolated from PDAC tissue of *Kras*^*LSL−G12D/+*^*Ink4a*^*fl/fl*^*Ptf1a*^*Cre/+*^ (*KIC*) mice. Next, we conducted an interactive analysis and exploration of cell-cell communication enabled by CellChat. We identified five targetable ligand combinations which specifically possess a kinase domain, an extracellular domain, or being secreted in the extracellular compartment (Fig. 3a, b). Further screening revealed hepatocyte-derived MIF expression correlated with fatty liver severity, validated by IHC/mIHC (Fig. 3c, d, Supplementary Fig. 3a-e). In addition, the majority of MIF were secreted by hepatocytes as shown by co-localization of MIF with albumin and F4/80 (Supplementary Fig. 3e).

To test whether hepatocyte-derived MIF is a key factor mediating fatty liver metastasis, we first investigated the source of MIF under the MASLD condition by directly isolating extracellular vesicles (EVs) from MASLD livers. After confirming the purity of the EVs (Supplementary Fig. 3f), we tested for MIF expression by Western Blot (Supplementary Fig. 3g). Our findings indicate that, consistent with previous studies,^23^ MASLD-derived MIF was predominantly released in the form of EVs.

CellChat analysis nominated CD44 as the dominant MIF receptor on PDAC cells (Fig. 3b), corroborated by immunofluorescence in KPC cells (Fig. 3e). CD44, a glycoprotein regulating tumor stemness and adhesion,^35^ was functionally tested using recombinant MIF (rMIF). Dose-dependent rMIF treatment enhanced transmigration in both KPC and AsPC-1 cells but did not affect proliferation (Fig. 3f and Supplementary Fig. 3h-j). *Cd44* silencing abolished this chemotactic response in KPC cells (Fig. 3g), demonstrating that the chemotactic stimulation of KPC cells by MIF predominantly operates via the CD44 pathway.

To explore the biological characteristics of metastatic pancreatic cancer within the context of fatty liver, we conducted a bulk RNA-seq analysis of metastatic lesions and performed Gene Set Enrichment Analysis (GSEA) and analyzed Kyoto Encyclopedia of Genes and Genomes (KEGG) pathways based on the sequencing results. KEGG pathway enrichment revealed upregulation of focal adhesion and proteoglycans in cancer pathways—the latter featuring CD44 as a key molecule involved in MASLD-associated metastases (Data S3 and Supplementary Fig. 3k, l). GSEA further highlighted CD44’s involvement in tumor stemness pathways, consistent with enhanced aggressiveness under fatty liver conditions (Data S4-5 and Supplementary Fig. 3m, n). Therefore, based on murine models, we proposed that liver metastatic foci in mice with combined fatty liver exhibit enhanced MIF-CD44 expression, which is accompanied by enhanced tumor stemness and adhesion capabilities.

### Hepatic MIF knockdown attenuates MASLD-associated pancreatic cancer stemness and metastatic adhesion and alters TAM spatial dynamics via CD44-mediated mechanisms

**Fig. 4:**
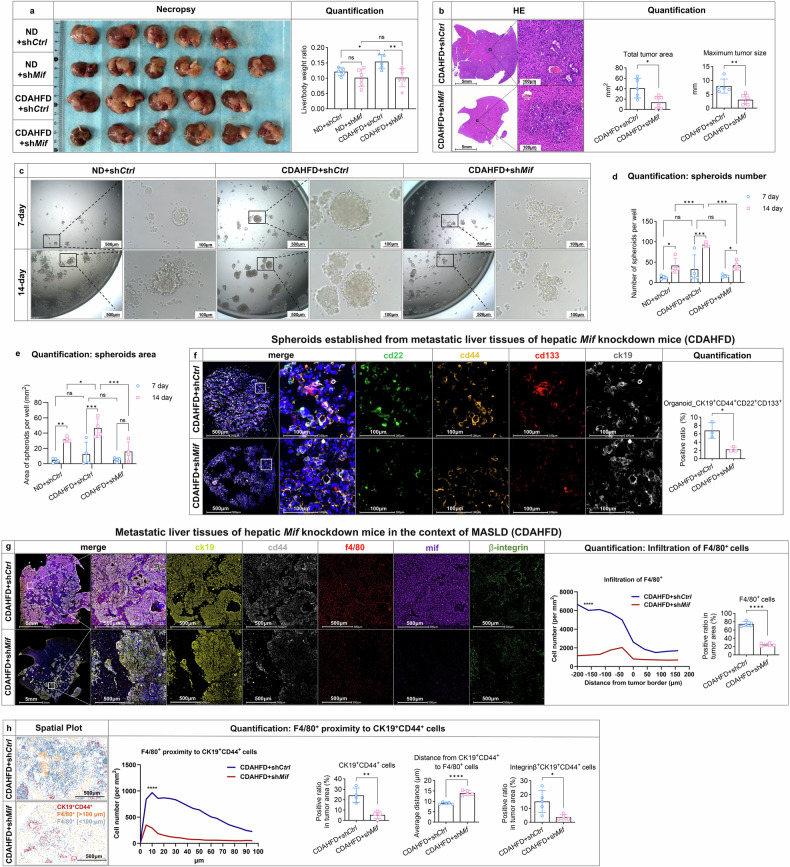
Hepatic MIF knockdown attenuates MASLD-associated pancreatic cancer stemness and metastatic adhesion and alters TAM spatial dynamics via CD44-mediated mechanisms. AAV_sh*Ctrl* or AAV_sh*Mif* virus were injected through the tail vein of the mice for 4 weeks to achieve hepatic MIF knockdown, followed by an ND or CDAHFD for another 4 weeks. Four groups of mice were included: (1) ND+sh*Ctrl*; (2) ND+sh*Mif*; (3) CDAHFD+sh*Ctrl*; (4) CDAHFD+sh*Mif*. Mice were injected with KPC cells in the spleen and sacrificed on day 15 (n = 5–6). **a** Representative macroscopic appearance of the liver metastases and the liver to body weight ratio in four groups. **b** HE staining of liver metastases in the CDAFHD-fed groups with quantification of maximum tumor size and the number of metastatic foci. **c** Sphere formation assay was performed on metastatic tissues from ND+sh*Ctrl*, CDAHFD+sh*Ctrl*, and CDAHFD+sh*Mif* groups, respectively. 3000/well were seeded in 96-well ultra-low attachment plates in stem cell medium. 7 days later, the spheroids were resuspended and seeded again (n = 4). **d**, **e** Quantification of number and size of spheres 7 days after each seeding. **f** Sphere formation assay was performed on metastatic tissues from ND+sh*Ctrl*, CDAHFD+sh*Ctrl*, and CDAHFD+sh*Mif* group, respectively. 3000/well were seeded in 96-well ultra-low attachment plates in stem cell medium. 7 days later, the spheroids were resuspended and seeded again (n = 3). A 4-color mIHC staining including CD22, CD133, CD44 and CK19 was performed on spheroids collected after 14 days of cultivation in CDAHFD+sh*Ctrl* and CDAFHD+sh*Mif* groups, respectively. Representative images, including whole slide, merged channels and separate channels were shown. The total number of CD22^+^CD133^+^CD44^+^CK19^+^ cells in the spheroids were quantified by Halo software using HighPlex FL v4.2.14 module in the column chart. **g** A 6-color mIHC staining, including CK19, CD44, F4/80, MIF, integrinβ was performed on metastatic liver tissues from CDAHFD+sh*Ctrl* and CDAHFD+sh*Mif* groups, respectively. Representative images including whole slide, merged channels and separate channels were shown. Spatial plot of F4/80^+^ cells was performed on metastatic liver tissues from CDAHFD+sh*Ctrl* and CDAHFD+sh*Mif* groups, respectively. The total number of F4/80^+^ cells in the tumor area were quantified by Halo software using HighPlex FL v4.2.14 module in the column chart (n = 5). **h** Spatial plot between CD44^+^CK19^+^ cells and F4/80^+^ cells were performed on metastatic liver tissues from CDAHFD+sh*Ctrl* and CDAHFD+sh*Mif* groups, respectively. CD44^+^CK19^+^ cells were shown as red dots. F4/80^+^ cells located more than 100 μm away from CD44^+^CK19^+^ cells appear in blue, while those within a 100 μm distance are depicted in gray. Proximity analysis and nearest neighbor analysis were performed on CD44^+^CK19^+^ cells and F4/80^+^ cells (n = 5), and the total number of CD44^+^CK19^+^ cells and integrinβ^+^CD44^+^CK19^+^ cells in tumor areas were quantified by Halo software using HighPlex FL v4.2.14 module (n = 4–5). Data are shown as mean ± SD per group. Unpaired parametric Student’s *t*-test or one-way ANOVA was performed to identify differences between two groups or among different groups, respectively. For proximity analyses, two-way ANOVA followed by Šídák’s multiple comparison test was performed to identify differences between two groups at different distances. A *p*-value less than 0.05 was considered statistically significant. *p < 0.05, ** *p* < 0.01, *** *p* < 0.001, and **** *p* < 0.0001; “n.s.” indicates not significant

To investigate whether fatty liver-derived MIF promote liver metastasis in vivo, we generated liver-specific knockdown of *Mif* using the AAV8 system, which enables precise manipulation of gene expression primarily in hepatocytes within the liver.^36^ To specifically investigate the role of MIF in metastasis without confounding its potential effects on MASLD development, we injected AAV8_sh*Mif* at the time of fatty liver establishment (4 weeks after CDAHFD feeding). Four weeks after *Mif* silencing, *Mif* mRNA was decreased; its low production level was maintained until the end of the experiment (Supplementary Fig. 4a, b). Metastatic liver tumor growth was significantly augmented in mice with MASLD but was noticeably inhibited upon *Mif* silencing (Fig. 4a, b). A similar inhibitory effect was observed in the control group, although to a lesser extent (Fig. 4a).

To establish conclusive evidence regarding the role of MIF in promoting cancer stem-like properties, we conducted experiments using tumor tissues obtained from three distinct groups: the ND group, the CDAHFD group, and the CDAHFD group with hepatic *Mif* knockdown. We cultured these tissues into organoids and assessed their self-renewal abilities through spheroid formation experiments and serial replating assays. Our findings revealed significant increases in both spheroid size and number in the CDAHFD group. Conversely, knocking down hepatic MIF caused fragmentation of these spheroids and the appearance of ghost-like cells, resulting in a corresponding reduction in spheroid size and number (Fig. 4c-e).

Since CD44 has been identified as a reliable marker for enriching cancer stem cells (CSCs) and it’s co-expression with other markers in the regulation of stemness and metastasis in various malignancies, including pancreatic cancer,^37,38^ we next collected the spheroids (after second plating) and stained with stemness markers (including CD44, ALDH1, and KLF4).^39,40^ The results showed that the expressions of stemness markers consistently mirrored spheroid formation capabilities in the above three groups (Supplementary Fig. 4c). In addition, multi-marker analysis revealed significant reductions in CD44⁺CD133⁺CD22⁺ co-positive cell populations within liver metastases of *Mif* knockdown mice compared to controls (Fig. 4f). Collectively, these findings demonstrate that hepatic MIF depletion attenuates CSC-associated stemness properties in the context of fatty liver conditions.

We next assessed the impact of hepatic *Mif* knockdown on key immune components within the metastatic microenvironment. MIF depletion significantly reduced MDSC infiltration while concomitantly increasing CD8⁺ T cell abundance (Supplementary Fig. 4d, e). Furthermore, total TAM infiltration was markedly decreased, with reductions in both M1 and M2 TAM subsets (Fig. 4g, Supplementary Fig. 4f). In contrast, Treg frequencies remained unchanged (Supplementary Fig. 4e). Analysis of TAMs spatial distribution revealed a significant decrease in infiltration specifically within the central tumor regions (Fig. 4g).

On the basis of the relevant differences in the TAMs and cancer cell stemness, we then conducted a spatial analysis to examine the distances of TAMs in relation to CD44^+^ cancer cells. Hepatic *Mif* knockdown not only reduced the overall infiltration of TAMs and the number of CD44^+^CK19^+^ cancer cells, but TAMs existed in a more distant proximity to CD44^+^ cancer cells as compared to the control group (Fig. 4g). In addition to stemness signature, CD44-initiated adhesion orchestrates the expression and activation of β1-integrin receptors, promoting the interaction of β1 integrins with extracellular matrix (ECM) proteins, thereby facilitating cell mobility in multiple tumors.^41,42^ We also found that a subset of integrinβ^+^CD44^+^ cancer cells were significantly reduced in hepatic *Mif* knockdown group (Fig. 4h). In summary, these results indicate that hepatic *Mif* knockdown may result in improved hepatic immune microenvironment, reduced cancer stemness and adhesion in MASLD, which was partially dependent on CD44.

### Pretreatment of MIF inhibitor or therapeutic targeting of MIF-CD44 axis inhibits metastatic tumor burden in orthotopic and metastatic models

**Fig. 5:**
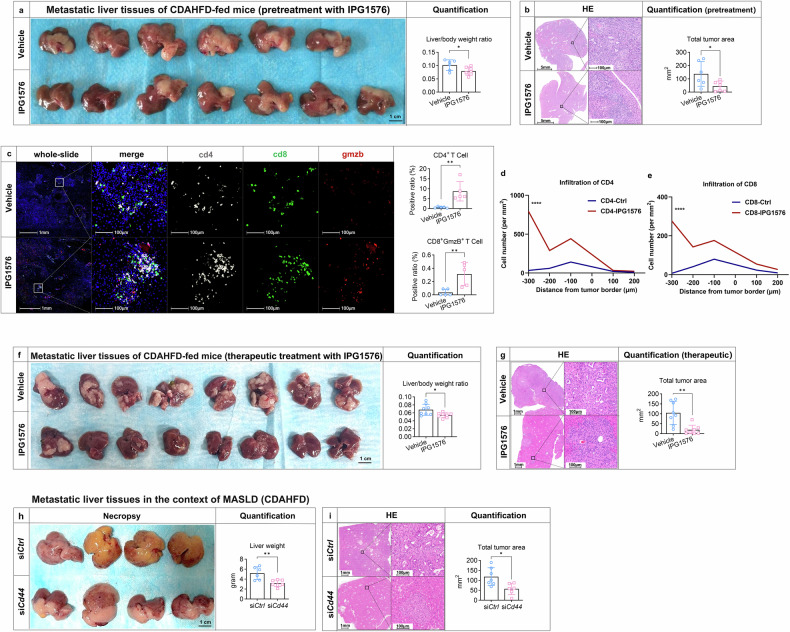
Pretreatment of MIF inhibitor or therapeutic targeting of MIF-CD44 axis inhibits metastatic tumor burden in orthotopic and metastatic models. Mice were fed with a CDAHFD for 4 weeks before the establishment of the liver metastasis model. The compound IPG1576 (30 mg/kg/d) was administered twice daily via oral gavage and commenced 7 days intrasplenic injection of 10^6^ KPC cells and continued for a duration of 14 days. An equivalent quantity of solution, 5%DMSO and 95% (20% [2-Hydroxypropyl]-β-cyclodextrin), was used as vehicle for IPG1576 (n = 6–7). **a** Representative macroscopic appearance of the liver metastases and the liver to body weight ratio in two groups. **b** HE staining of liver metastases in the MASLD groups with quantification of maximum tumor size and the total area of metastatic foci. **c** Tumor microenvironment of liver metastasis was examined by mIHC staining of 3-color mIHC staining including CD4, CD8 and GmzB on metastatic liver tissues from vehicle and IPG1576 group, respectively. Representative images including whole slide, merged channels and separate channels were shown. The ratio of CD4^+^ T cells and GmzB^+^CD8^+^ T cells were quantified by Halo software using HighPlex FL v4.2.14 module (n = 5). **d**, **e** The infiltrated cell number of CD4 and CD8 per mm^2^ tissue across the tumor border was quantified (n = 5). **f** Mice were fed with a CDAHFD for 4 weeks followed by the establishment of the liver metastasis model. Five days after intrasplenic injection of KPC cells (5×10^5^), IPG1576 (30 mg/kg/d) was administered twice daily for a duration of 14 days (n = 8). Representative macroscopic appearance of the liver metastases and the liver to body weight ratio were shown. **g** HE staining of liver metastases in the MASLD groups with quantification of the total area of metastatic foci (n = 8). **h** Mice were fed with a CDAHFD for 4 weeks followed by the establishment of the liver metastasis model using KPC cells (10^6^) transfected with si*Ctrl* or si*Cd44* (n = 6–7). Representative macroscopic appearance of the liver metastases in the si*Ctrl* and si*Cd44* groups were shown and liver weight was measured. **i** HE staining of liver metastases in the si*Ctrl* and si*Cd44* groups with quantification of the total area of metastatic foci (n = 6–7). Scale bar was indicated in the individual figures. Representative pictures are shown. Data are shown as mean ± SD per group. Unpaired parametric Student’s *t* test was performed to identify differences between the two groups. For proximity analyses, two-way ANOVA followed by Šídák’s multiple comparison test was performed to identify differences between two groups at different distances. A *p* value less than 0.05 was considered statistically significant. *p < 0.05, ** *p* < 0.01, *** *p* < 0.001, and **** *p* < 0.0001; “n.s.” indicates not significant. GmzB, granzyme B

IPG1094 is a clinical-stage small molecule MIF inhibitor currently being evaluated in Phase I trials for advanced solid tumors. IPG1576, a next-generation compound derived from the same platform, demonstrates improved pharmacokinetic properties and target selectivity relative to IPG1094 (Data S6). We first assessed the effects of IPG1576 pretreatment in the context of MASLD, administering IPG1576 seven days before establishing the liver metastasis model. IPG1576 administration significantly reduced liver metastasis in the setting of MASLD, as demonstrated by necropsy, liver/body weight, and total tumor area (Fig. 5a, b). Consistent with results of hepatic *Mif* knockdown, MIF inhibitor IPG1576 significantly reduced the number of TAMs in the center and the border of the tumor. However, IPG1576 treatment did not alter the M1/M2 TAM polarization ratio (Supplementary Fig. 5a-c). In addition, pretreatment with IPG1576 not only increased the number of CD4^+^ and cytotoxic CD8^+^ cells (Fig. 5c) but also altered the distribution of T cells within the tumor, promoting greater infiltration into the tumor core rather than the periphery (Fig. 5d, e). MDSC infiltration was significantly decreased by IPG1576 while Treg abundance remained unaltered (Supplementary Fig. 5d, e).

Furthermore, MIF inhibition in MASLD led to a reduction in integrinβ^+^CD44^+^ cancer cells. Spatial analysis revealed there was more distant proximity between TAMs and CD44^+^ cancer cells upon MIF inhibition (Supplementary Fig. 5f). In terms of cancer cell stemness, MIF inhibition in MASLD led to a reduction in CD44^+^ or ALDH^+^ or CD44^+^/KLF4^+^ cancer cells (Supplementary Fig. 5g). Beyond stemness markers, IPG1576 pretreatment also increased tumor cell apoptosis without affecting proliferation in MASLD livers (Supplementary Fig. 5h). These results suggest that pretreatment of MIF inhibitor in MASLD effectively ameliorates PDAC hepatic metastases.

In addition to pretreatment effects, we also assessed the therapeutic efficacy of IPG1576 in the liver metastasis model in both ND- and CDAHFD-fed mice. In alignment with hepatic MIF knockdown, the effects of IPG1576 were more pronounced under MASLD conditions, highlighting the potential for personalized treatment in the subset of PDAC patients with MASLD (Fig. 5f, g, Supplementary Fig. 5i).

The intrasplenic model is highly efficient at inducing liver metastasis; however, it lacks the physiological and clinical relevance of the orthotopic model. Therefore, we next employed the orthotopic model for the application of IPG1576. MASLD induced a higher incidence of liver metastases compared to Ctrl mice (70.6% vs. 33.3%; data combined from batch-1 [Supplementary Fig. 2d] and batch-2 [Supplementary Fig. 5j]), whereas IPG1576 demonstrated specific inhibitory effects on liver metastases only in the MASLD group (CDAHFD group, IPG1576 70.6% vs. vehicle 30%; ND group, IPG1576 33.3% vs. vehicle 40%)

To confirm the functional role of CD44 in metastasis within the MASLD context, we next knocked down CD44 expression in KPC cells and injected them into the spleen to establish liver metastasis. Critically, CD44 knockdown significantly attenuated liver metastasis progression. This attenuation was evidenced by a marked reduction in metastatic tumor area and a concomitant decrease in liver weight compared to the control group injected with non-targeting siRNA-transfected cells (Fig. 5h, i). Collectively, these findings demonstrate CD44’s essential role in promoting liver metastasis in MASLD. Therefore, therapeutic strategies targeting the MIF-CD44 axis, such as pretreatment with a MIF inhibitor, hold significant translational potential under MASLD conditions.

### MIF-CD44 expression pattern in PDAC patients with MASLD

**Fig. 6:**
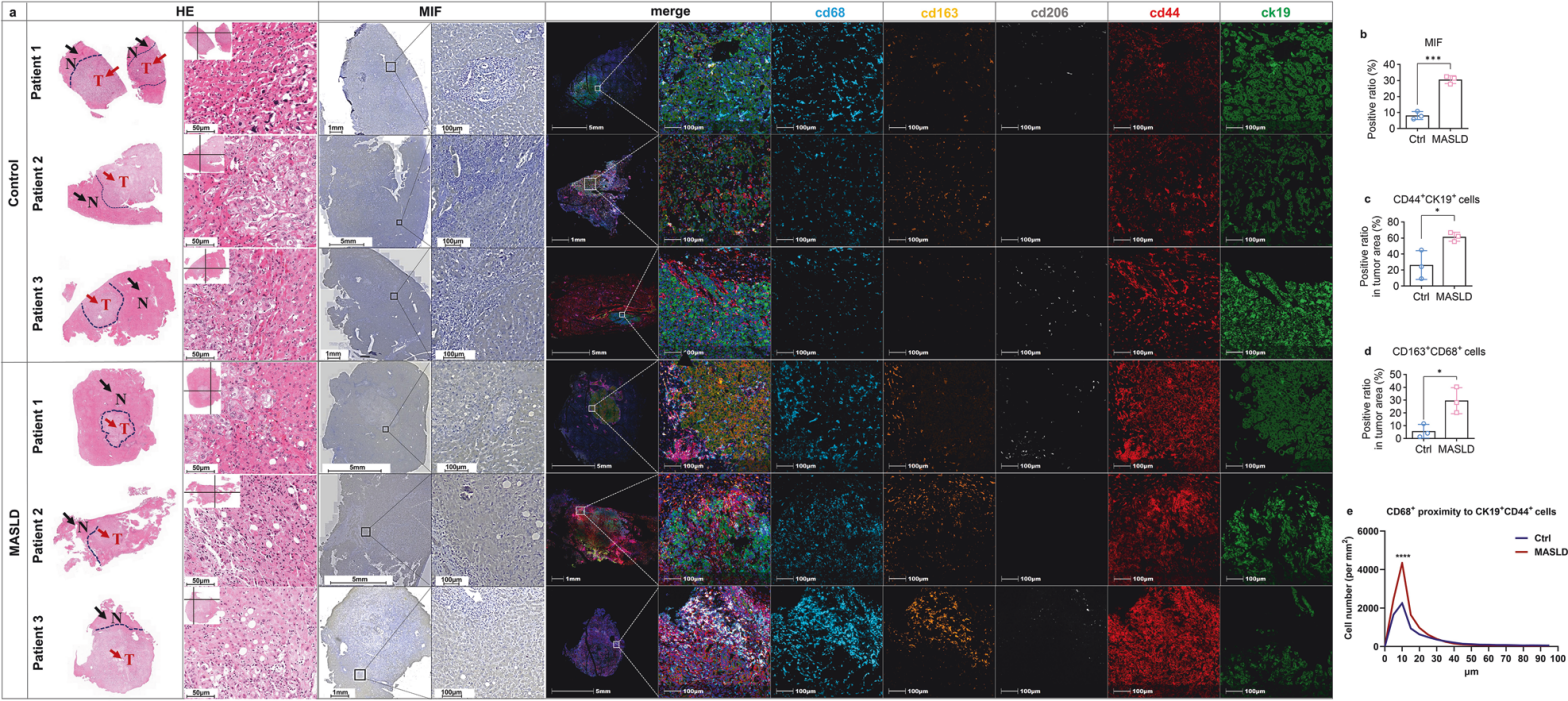
MIF-CD44 expression pattern in PDAC patients with MASLD. **a** Metastatic PDAC tissues from patients were stained with HE, MIF by IHC, and a 5-color panel including CD68, CD163, CD206, CD44, CK19 by mIHC (n = 3 for Ctrl group and MASLD, respectively). Tumor area was indicated by red arrow and non-tumor area was labeled by black arrow on HE slides. **b** The expression of MIF was assessed by Image J based on whole-slide scanning and quantification. **c**, **d** The ratio of CK19^+^CD44^+^ cells and CD163^+^CD68^+^ cells were quantified by Halo software using HighPlex FL v4.2.14 module based on whole-slide scanning. **e** Proximity analysis between CD44^+^CK19^+^ cells and CD68^+^ cells were performed on the whole slide of metastatic liver tissues from PDAC patients using Halo software. Data are shown as mean ± SD per group. Unpaired parametric Student’s *t*-test was performed to identify differences between two groups. For proximity analyses, two-way ANOVA followed by Šídák’s multiple comparison test was performed to identify differences between two groups at different distances. A *p*-value less than 0.05 was considered statistically significant. *p < 0.05, ** *p* < 0.01, *** *p* < 0.001, and **** *p* < 0.0001

Given the infrequent surgical resection for PDAC patients with liver metastases and that obtaining large liver tissue samples is challenging, we collected a limited number of samples from liver oligometastases in individuals with pancreatic cancer. These samples were then subjected to IHC and mIHC validation and spatial analysis. Consistent with experimental models, liver tissues from MASLD patients with hepatic metastases exhibited significant upregulation of MIF in non-tumoral parenchyma and CD44 expression in tumor cells (Fig. 6a-c). Additionally, mIHC staining revealed an increase in M2-type TAMs positive for CD163 and CD68 within the tumor regions, whereas CD206 was poorly expressed, indicating that CD163 was a better marker in this scenario (Fig. 6d). Notably, compared to the control group, the number of total TAMs were not significantly different but were found to be in closer proximity to CD44^+^ tumor cells with a stem-like phenotype (Fig. 6e). These findings corroborate previous results obtained in animal models, highlighting the specific expression pattern of the MIF-CD44 axis and its associated spatial distribution in liver metastasis patients with concurrent MASLD, making it a promising target for tailored interventions in the context of PDAC, suggesting that inhibition of MIF-CD44 axis has translational potential.

## Discussion

This study provides compelling evidence that MASLD significantly accelerates PDAC progression and liver metastases through the MIF-CD44 axis. By leveraging large-scale epidemiological data, clinical cohorts, patient tissues and in vitro and in vivo preclinical models, we demonstrate that MASLD fosters a permissive microenvironment for metastatic seeding and growth, driven by enhanced tumor cell pluripotency, immune evasion, and adhesion. These findings have important implications for understanding the interplay between metabolic disorders and cancer progression. Our epidemiological analysis of the UK Biobank dataset revealed a striking increase in PDAC risk within MASLD population. In addition, PDAC patients with concurrent MASLD are more likely to develop metastasis and experience a faster progression of liver metastases in three clinical cohorts. Animal models further validate the cause-and-effect relationship between fatty liver and liver metastasis. These observations underscore the need for heightened surveillance and early intervention in PDAC patients with MASLD.

Mechanistically, we identified MIF as a central mediator of MASLD-induced metastasis. MIF secretion by steatotic hepatocytes promotes the migration and adhesion of CD44-positive PDAC cells, enhancing their stemness and metastatic potential. This discovery extends previous evidence of tumor-derived MIF priming premetastatic niches by uncovering a novel hepatocyte-tumor cell crosstalk.^23^ Preclinical models validated the pro-metastatic role of MIF, with genetic knockdown or pharmacological inhibition of MIF significantly reducing liver metastasis. Critically, the efficacy of MIF inhibitor IPG1576 is strictly MASLD-dependent: it significantly attenuated metastasis only under MASLD conditions in both splenic injection (Fig. 5a, f) and orthotopic models (Supplementary Fig. 5j). Similarly, liver-specific *Mif* knockdown was more effective at reducing liver metastasis in mice with MASLD-associated liver metastasis (Fig. 4a). In addition, the MIF tautomerase inhibitor IPG1576 not only decreased metastatic tumor burden but also restored immune surveillance by enhancing CD8^+^ T cell infiltration and reducing TAM density within metastatic lesions. This finding is consistent with the established role of IPG1576 in suppressing MDSC differentiation, further extending its therapeutic potential to MASLD-driven metastasis.^43^ These findings highlight the dual role of MIF in modulating tumor cell behavior and the immune microenvironment. Thus, our research offers novel insights and lays the groundwork for personalized treatment strategies in pancreatic cancer patients with concurrent liver conditions.

Validation in patient-derived metastatic liver tissues confirmed elevated hepatic MIF expression and increased CD44-positive tumor cells in MASLD-associated PDAC cases. The spatial proximity between TAMs and CD44-positive cancer cells further supports the role of MIF-CD44 signaling in shaping the metastatic niche. This spatial reorganization is consistent with the “seed and soil” paradigm observed in MASLD-driven colorectal cancer metastasis, wherein hepatocyte-derived EVs remodel the tumor-stroma interactions.^44^ These translational findings underscore the clinical relevance of targeting the MIF-CD44 axis in MASLD-associated PDAC.

Despite its strengths, our study has limitations. The precise cellular sources of MIF, as well as the potential roles of MIF in angiogenesis within the liver, were not fully delineated, and further studies using cell-type-specific knockout models are warranted. While MIF is necessary for MASLD-accelerated metastasis, it is insufficient to drive metastasis to the extent observed in the complex MASLD microenvironment (data not shown), indicating metastatic progression involves multifactorial mechanisms in addition to MIF. Although our preclinical models recapitulate key features of MASLD-induced metastasis, they may not fully capture the complexity of human disease. Recent advances in hepatic epithelialization mechanisms highlight the importance of liver-derived signals in metastatic colonization, providing a broader context for our findings.^16^ Future research should explore the role of other hepatic secreted factors and their interactions with systemic metabolic dysregulation in PDAC progression. Critically, we acknowledge that functional immune profiling (e.g., checkpoint molecules such as PD-1 or CTLA-4) was not performed in this study. While our spatial analyses revealed exclusion of CD8^+^ T cells from tumor cores and increased M2-like TAMs in MASLD-associated metastases, these data do not directly interrogate the functional state of immune cells. While beyond our current scope, future work should integrate single-cell transcriptomics with functional assays to determine how hepatic MIF reprograms immune cell activity in the metastatic niche. High-resolution spatial transcriptomics will dissect MIF–CD44 signaling within specific niches, enabling mechanistic interpretation of their spatial and cellular context.

Analysis of the liver metastatic TME revealed concurrent expansion of both M1-polarized and M2-polarized TAM populations in metastases from CDAHFD-induced MASLD mice. Hepatic *Mif* knockdown or MIF inhibitor (IPG1576) treatment both induced a reduction in total TAM infiltration; however, MIF knockdown significantly decreased both TAM subsets, whereas IPG1576 had no effect on M1/M2 polarization. This divergence might arise from genetic ablation eliminating hepatic MIF, whereas pharmacological inhibition allows compensatory signaling pathways and non-receptor-mediated MIF functions.^45,46^ In addition, these findings suggest the functional plasticity and spectrum of activation states inherent to TAMs within the metastatic niche, transcending a strict M1/M2 dichotomy.^47,48^ Future studies should focus on expanding patient-derived sample cohorts to further delineate MASLD-associated TAM subtypes and functions in human metastatic liver disease.

In this study, we employed three complementary dietary models: MCD, CDAHFD, and long-term HFHCD. Notably, these models exhibit distinct advantages and limitations: The MCD and CDAHFD rapidly induce histopathological features resembling human MASH (steatohepatitis and fibrosis) but lack obesity and insulin resistance, whereas the HFHCD model captures systemic metabolic syndrome at the expense of prolonged induction time. Consequently, while all models confirmed the pro-metastatic role of MASLD, the observed effects in MCD/CDAHFD models primarily reflect the impact of the hepatic microenvironment (e.g., MIF secretion and stromal remodeling), whereas the HFHCD model, encompassing the systemic metabolic dysregulation characteristic of human MASLD, including obesity, insulin resistance, and adipose tissue inflammation, suggests that these broader metabolic alterations may independently influence tumor progression and metastatic behavior through endocrine signaling or systemic immune modulation.

While our study identifies the MIF-CD44 axis as a key driver of MASLD-associated PDAC liver metastasis, we acknowledge that MIF also interacts with receptors such as CD74, CXCR2, and CXCR4, which may contribute to the metastatic microenvironment, particularly in immune cells like TAMs and MDSCs.^17,46^ For instance, MIF-CD74 signaling could enhance M2-like TAM polarization, while MIF-CXCR2/CXCR4 interactions may promote immune cell recruitment or tumor cell homing.^46^ Additionally, CD44 ligands such as hyaluronic acid, osteopontin, and MMPs,^35^ which were not prioritized in our screening, may amplify CD44-mediated adhesion and invasion in the MASLD liver. Our in vitro experiments demonstrate that MIF acts as a chemoattractant for pancreatic KPC cells. Notably, this chemotactic response requires CD44 receptor expression on KPC tumor cells. However, the absence of CD74 expression on KPC cells-induced metastatic liver tissues (Data not shown) suggests that MIF-CD44 is the dominant pathway in PDAC cells, but the broader signaling network likely involves crosstalk with these alternative pathways. Future studies using cell-type-specific knockouts, proteomic profiling, or ligand-specific inhibitors will be critical to fully elucidate these interactions and refine therapeutic strategies targeting the metastatic niche in MASLD-associated PDAC.

Metastasis is nearly universal in PDAC, with recurrence occurring in almost all patients after resection, contributing to poor prognosis. While our study identifies MASLD as a key accelerator of liver metastasis via the MIF-CD44 axis, this mechanism may extend beyond MASLD-positive patients. Clinically, the high prevalence of MASLD (25.7% in our cohort, rising to 53.81% in those with liver metastases) defines a large subgroup with accelerated progression. Targeting the MIF-CD44 may be particularly impactful for these patients, especially given their younger age at diagnosis and poorer survival. In the broader PDAC population, metastasis is driven by tumor-intrinsic factors, systemic immune dysregulation, and organ-specific microenvironments. Therefore, therapeutic strategies should integrate broader approaches targeting other metastatic drivers to benefit most patients. For example, combining MIF inhibitors (e.g., IPG1576) with immunotherapies or KRAS-targeted therapies might offer synergy, particularly in patients with concurrent MASLD where the hepatic microenvironment is critical. Although IPG1576 efficacy was more pronounced with MASLD, baseline MIF/CD44 expression in non-MASLD PDAC suggests broader mechanistic relevance.

In conclusion, this study identifies the MIF-CD44 axis as a pivotal driver of MASLD-associated PDAC liver metastasis and provides a strong rationale for its therapeutic targeting. By addressing the unique challenges posed by metabolic comorbidities, our findings pave the way for personalized treatment strategies aimed at improving outcomes for PDAC patients with concurrent MASLD.

## Materials and methods

Detailed materials and methods, partial results and discussion regarding UK biobank, as well as figures and tables are described in the supplementary materials. Refer to Data S1 for all antibodies used in mIHC.

### Study design

The aim of our study is to assess the causal effect of MASLD on the progression of PDAC and liver metastasis. We began by analyzing a large prospective cohort from the UK Biobank and three retrospective clinical cohorts. We then established mouse models that mimic MASLD-induced hepatic metastasis, which showed increased tumor cell pluripotency and focal adhesion. Next, we conducted a single-cell RNA sequencing analysis of MASLD and PDAC samples from the GSE dataset. This was followed by a blind selection process to identify a specific ligand-receptor combination, MIF-CD44, notably associated with MASLD-induced attraction of tumor cells towards the liver. We examined the pro-metastatic role of MIF derived from fatty liver using AAV8-mediated gene knockdown and investigated whether these effects were mediated by CD44 on PDAC cells in vivo. The translational significance of this study was evaluated by targeting the MIF-CD44 axis either by a MIF inhibitor or by CD44 knockdown in various mouse models, and by validating this axis in human metastatic liver samples. All procedures, performed thrice, adhered to stringent ethical and scientific standards under the supervision of the animal censor at the Affiliated Hospital of Qingdao University.

### Retrospective cohorts and clinical samples

This study mainly includes three cohorts from Peking Union Medical College Hospital (BJ cohort), the Affiliated Hospital of Qingdao University (QD cohort), and Zhejiang Cancer Hospital (ZJ cohort) for retrospective research data, respectively. The retrospective analyses were conducted on computerized medical records to extract comprehensive clinical parameters in three cohorts (Supplementary Table 3). Contrast-enhanced computed tomography and/or magnetic resonance imaging were used to assess the number and size of metastatic sites in liver metastatic PDAC patients. The research excluded cases with uncommon malignant pancreatic illnesses, including non-ductal tumors (acinar, neuroendocrine, pancreatoblastoma, and solid pseudopapillary tumors), as well as secondary malignancies (e.g., ampullary, CBD cancers). The retrospective clinical study was conducted in accordance with the Ethical Review Approval for Clinical Research Projects by the Health Commission (MR-37-24-016655), including Ethical Committee of the Clinical Research Projects of Peking Union Medical College Hospital (I-24PJ0698), the Affiliated Hospital of Qingdao University (QYFYEC2024-50), and Zhejiang Cancer Hospital (IRB-2025-989), respectively.

For another research cohort, we performed H&E staining and mIHC on formalin-fixed paraffin-embedded (FFPE) tissues obtained from 6 PDAC liver metastasis patients at the Affiliated Hospital of Qingdao University. All cases of PDAC liver metastases were obtained from the Affiliated Hospital of Qingdao University from 2018 to 2020. The demographic and clinico-pathological features were shown in Supplementary Table 7. This cohort was conducted in accordance with the ethical guidelines established by the Affiliated Hospital of Qingdao University (QYFYWZLL28018).

### Animal models

#### Murine orthotopic and liver metastasis models

A preclinical, murine pancreatic tumor model of hepatic metastasis model was utilized in this study which closely resembles the clinical conditions observed in patients with liver metastases.^15^ Briefly, C57BL/6 male mice were injected intrasplenically with 5 × 10^5^ or 10^6^ KPC cells depending on the models in 25 µL of DMEM under isoflurane anesthesia. In the orthotopic model, mice were intra-pancreatic injected with 2.5 × 10^5^ KPC-luciferase cells suspended in 25 μL of DMEM. in vivo fluorescence imaging was performed on day 15 and 28 post-inoculation to monitor tumor metastasis. In the orthotopic model, mice were sacrificed on the 30th day.

#### Effect of IPG1576 on orthotopic and liver metastasis models

The compound IPG1576 (30 mg/kg/d) was administered orally via gavage twice daily, with each dose consisting of 300 μL and ensuring at least a 6-h interval between doses. In therapeutic models (both orthotopic and metastasis), the drug administration regimen commenced 5 days after the establishment of the metastasis model and continued for a duration of 14 days. In the pretreatment model, IPG1576 administration commenced seven days prior to the establishment of the metastasis model and continued for a duration of 14 days. An equivalent quantity of solution, 5% DMSO and 95% (20% [2-Hydroxypropyl]-β-cyclodextrin), was used as a vehicle for IPG1576. The recent study has detailed the absorption, distribution, metabolism, excretion, and preliminary safety analysis of IPG1576.^43^

#### MASLD induction

Wild-type (WT) mice (C57BL/6J background) were purchased from Charles River Laboratories (Beijing Vital River Laboratory Animal Technology Co., Ltd, China). They were housed under specific pathogen-free conditions with 12-h light–dark cycle and ad libitum access to water and food in the animal core facility of the Affiliated Hospital of Qingdao University. MASLD was induced using one of three diets, each selected for its ability to replicate distinct aspects of human MASLD pathophysiology:

- High Transfat (44 kcal%), High Cholesterol (2%), High Fructose (22%) Purified Rodent Diet (HFHCD) for 31 weeks (AMLN, Charles River Co., Ltd, China).
- Choline-deficient, L-amino acid-defined, high-fat diet (CDAHFD) for 4 weeks (TP3622657; Trophic Diets).
- Methionine- and choline-deficient diet (MCD) for 4 weeks (TP3005; Trophic Diets).

All animal experiments adhered to protocols approved by the Research Ethics Committee of the Affiliated Hospital of Qingdao University (AHQU-MAL20231113).

#### Mouse liver metastases organoid culture

Mouse liver metastasis tissues were removed from the preservation solution (Kingculture™, KCX-1) and rinsed with DPBS (Thermofisher, 17105041) three times. Tissues were cut into small pieces as much as possible with blood vessels, fat, necrosis being removed, and transferred to a clean centrifuge tube supplemented with Digestion Solution (Kingculture™, KCX) and placed in a water bath for 30 min at 37 °C at 140 rpm. After digestion, the tissues were rinsed with washing solution (Kingculture™, KCQ-1) for three times. During this period, tissue fragments were vortexed vigorously 10–20 times to obtain a homogenous suspension. The supernatant was then transferred to a new tube and centrifuged for 5 min at 1000 rpm. The solution was subsequently filtered through a 100 µm cell mesh sieve and centrifuged at 1000 rpm for 5 min. 4 × 10^5^ cells containing cancer cells and other TME cells and Matrigel (Corning, 356231) were mixed in a 200:1 ratio and inoculated into 6-well plates in 2 mL pancreatic cancer organoid medium (Kingculture^TM^ Organoid Growth Medium, KCW-M-8) per well. The medium was changed every 3 days.

After the organoids were constructed, they were broken down thoroughly and filtered through a 40μm filter to ensure that all cells are single cells. The diameter of the organoids should not be too large (preferably <400μm) to avoid long-term digestion, damage to cell activity, and subsequent cell sphere formation experiments.

#### Sphere formation assay

Liver metastasis organoids were seeded (3000 cells/well) in 96 well low-attachment culture plates in 75 µL of DMEM medium (Gibco). After cells were seeded into the plates, place the cells in a 6-hour incubator for starvation treatment to consume the cell factors related to the organoid culture medium that may remain in cells. After 6 h, add 2× pancreatic cancer tumor sphere culture medium (Kingculture™, KCW-M-13-100) in a 1:1 ratio for cultivation and observation. If cell clusters are found to be excessively aggregated, gently shake the well plate or use a wide-bore pipette tip to gently blow several times to help disperse the cells. Spheres were reseeded every 7 days to understand the self-renewal potential, and the number of spheres (>100 μm) for each well was evaluated after 7 and 14 days of cultivation. Each sample was seeded in 10 wells and images were taken daily for 14 consecutive days.

#### mIHC staining and analysis

mIHC was performed using the Opal 7-color fluorescent IHC Kit (PerkinElmer, Massachusetts, USA) according to the manufacturer’s instruction. In brief, 4-μm thick FFPE sections of murine or human metastatic tissues were deparaffinized and then fixed with 10% formalin fixation prior to antigen retrieval using citrate buffer (pH6) or Tris/EDTA (pH9) under high temperature and high pressure. Corresponding primary antibodies, secondary-HRP antibodies, and Opal TSA dyes were incubated with the slides for several rounds. After all sequential staining reactions, sections were counterstained with DAPI and mounted with antifade reagent (AR1109, BOSTER, Wuhan, China). The sequential multiplexed staining protocol is shown in Data S2. All mIHC slides were scanned using a Zeiss Axio Scan Z1 brightfield/fluorescence Slide Scanner at 20× magnification. For metastatic tissues obtained from patients with PDAC, whole-slide scan images were stained and analyzed. Hepatic metastatic tumor sections from mice were stained, and 3–5 representative fields were selected from whole-slide scan images for further analysis using HALO software (v3.6.4134, Indica Labs, USA) using Classifier (Random forest), HighPlex FL v4.2.14 module incorporated with Nearest neighbor analysis, Proximity analysis, and Infiltration analysis.

To differentiate tumor tissue from stroma, a tissue classifier leveraging the epithelial cell marker cytokeratin 19 (CK19) was utilized. The Highplex FL analysis algorithm facilitated cell phenotyping by defining nuclear detection sensitivity and minimum intensity thresholds based on staining localization (nuclear, cytoplasmic, or membrane). Sequentially, the counts of targeted cells were normalized to the total cell counts for the tumor areas to generate the density of positive cells per mm^2^ of tissue. Cell localization was assessed by measuring infiltration distances of different cell types along the tumor border. The proximity distance, representing the average number of TAMs within a 30-μm radius from the nuclear center of CD44^+^CK19^+^ tumor cells, was quantified using GraphPad Prism (v10).

## Supplementary information

Supplementary material

Data S1-S6

## Data Availability

All data are publicly available in the main text/supplementary materials or online database.
