# Supplementary material for: Metabolic dysfunction-associated steatotic liver disease accelerates pancreatic cancer progression and metastasis via the macrophage migration inhibitory factor-CD44 axis
Source: Signal Transduct Target Ther. 2026 Jan 16;11:32. doi: 10.1038/s41392-025-02562-8 (PMC12811260; doi:10.1038/s41392-025-02562-8)
Supplement: Supplementary file 1 — Supplementary material [file 41392_2025_2562_MOESM1_ESM.docx]

Supplementary Materials for

Metabolic Dysfunction-associated Steatotic Liver Disease Accelerates Pancreatic Cancer Progression and Metastasis via the migration inhibitory factor-CD44 axis

Qian Yu^1†^, Hui Song^1†^, Xiao-ya Shi^1†^, Liang Zhu^2†^, Yu Liang^1^, Rui-ning Gong^3^, Xiao-wu Dong^4^, Shang-long Liu^5^, Hai-zhen Wang^1^, Ying-luo Wang^6^, Jiu-fa Cui^7^, Xiao-nan Yang^7^, Ying Chen^6^, Chao Gao^1^, Zhan Yang^1^, Qing-tian Zhu^4^, Chang Li^8^, Huan Zhang^9^, Jie-er Ying^10^, Mei-fang Zheng^10^, Yan-tao Tian^11^, Hai-tao Hu^11^, Xin-xin Shao^11^, Yue Li^12^, Ming-guang Mo^13^, Yun Lu^5^, Zheng Ma^5^, Shun-li Fu^1^, Qing-hui Niu^14^, Yuan-yu Liao^1^, Chen-yang Zhao^1^, Xin Liu^15^, Ashok K. Saluja1^1,16^, Ji-gang Wang^17^, Xiao-yu Li^18^, Song-yue Guo^1^, Wei-hua Wang^1^, Song Wang^5^, Bin Liu^2^, Guo-tao Lu^4*^, He Ren^1,3*^

Correspondence to: herenrh@163.comxxxxx@xxxx.xxx

**This PDF file includes:**

Materials and Methods

Supplementary Text

Figures S1 to S6

Tables S1 to S15

Captions for Data S1 to S6

**Other Supplementary Materials for this manuscript include the following:**

Data S1 to S6

Data S1 All antibodies were listed in Data S1.

Data S2 The mIHC staining panels were listed in Data S2.

Data S3 KEGG analysis of upregulated pathways in MASLD group (n=3 per group)

Data S4 GSEA analysis listing all gene sets enriched in phenotype MASLD group (n=3 per group)

Data S5 Gene sets associated with pluripotency in MASLD group (n=3/group)

Data S6 IPG1576 profile summary

Materials and Methods

Definition of MASLD (retrospective cohorts)

Patients included in this study who were diagnosed with pancreatic cancer underwent abdominal computed tomographic (CT) scanning at the time of initial diagnosis in the PU cohort. The CT diagnosis involved using unenhanced CT scanning to measure the Hounsfield unit (HU) values of the liver and spleen. The liver-to-spleen (L/S) ratio of HU <1.0 was utilized for diagnosing and assessing the severity of liver fat content.^1^ The study excluded individuals with liver conditions like chronic viral hepatitis B/C, autoimmune hepatitis, primary biliary cholangitis, or liver cirrhosis. Additionally, those who consumed excessive alcohol (≥30 g/day for males and ≥20 g/day for females) or had a history of hepatocellular carcinoma or other liver-related malignancies were not considered for participation.

Extracellular vesicle extraction

Mice were dipped in 75% ethanol for 10s twice. The liver was perfused by 50 ml sterile perfusion fluid via postcava to remove red blood cells and granulocytes. To remove blood, mouse livers were flushed by perfusion with PBS through inferior vena cava in situ before collecting the whole liver. EVs were isolated from culture supernatant as follows. The medium was centrifuged at 300 ×g for 10 minutes to remove whole cells and then at 2000 ×g for 10 minutes to remove debris and apoptotic bodies. The resulting medium was applied to a 0.22 μm vacuum filtration unit (Sigma-Aldrich, Cat# S2GPU05RE) to eliminate large EVs, and then the supernatant was centrifuged at 100,000 ×g for 70 minutes. The final pellet was resuspended in PBS.

Production of AAV particles and in vivo AAV vector treatment

For hepatic *Mif* knockdown, three unique shRNA constructs targeting *Mif* (sh*Mif* #1, #2 and #3) and a nontargeting scrambled control (sh*Ctrl*) cloned into pLKO.1-EGFP-puro vector were used in this study. Knockdown of *Mif* was confirmed by transfection in AML12 cell followed by Western blotting. Construct #2 was then subcloned into AAV vector (AAV-U6-MCS-CMV-ZsGreen) for subsequent production of AAV particles. The AAV_sh*Ctrl* virus was used as a negative control. All plasmids were confirmed by restriction enzymes and sequencing. AAV particles were purified, tittered, and injected through mice with a titre of 10^11^ four weeks prior to the establishment of the hepatic metastasis model. For the viral packaging procedure, the triple plasmid adeno-associated virus system—which consists of the pAdDeltaF6 plasmid, AAV serotype 8, and plasmid containing target gene vectors—was employed. Using the HighFectin transfection reagent, the three plasmids were co-transfected with 293T cells following high purity endotoxin-free extraction of each plasmid vector. The precipitates of the cells were obtained 72 hours following transfection. Viral titre, mycoplasma, and sterility tests were conducted as part of the quality control process for viruses. The packaging, purification, and titration of the reassembled viral vector were carried out by Shanghai Integrated Biotech Solutions (Shanghai, China).

In vivo imaging

Prior to imaging, mice were anesthetized using a laboratory animal anesthesia system (R546Pro, RWD Life Science) with 2-3% isoflurane in oxygen. D-luciferin potassium salt (SAITONG, Cat# L10060) was administered via intraperitoneal injection at a dose of 10 μL per gram of body weight, prepared as a stock solution of 15 mg/mL in sterile PBS. At 5 minutes post-injection, the mice were placed in the light-tight chamber of the NightOWL II LB983 small animal in vivo imaging system. Anesthesia was maintained throughout the imaging procedure with continuous delivery of 1.5-2% isoflurane. Grayscale photographic images and bioluminescence images were acquired and overlaid using the system's native software (IndiGO) to localize the source of the bioluminescence signal.

Single-cell preparation from liver metastatic tissue and staining for flow cytometry

Mice were euthanized, and liver metastases were harvested. Tissues were minced and enzymatically digested for 30 minutes at 37°C in a solution containing 0.04 mg/mL collagenase I (Worthington Biochemical, LS004194), 1.31 mg/mL collagenase IV (Worthington Biochemical, LS004188), and 0.015 mg/mL DNase I (Macklin, D806930). The digestion was quenched with fetal bovine serum (FBS), and the resulting cell suspension was filtered through a 100 µm nylon mesh. To enrich for immune cells, the suspension was layered over a discontinuous 40%/80% Percoll gradient and centrifuged at 800 × g for 30 minutes with brake disabled. The immunocyte-enriched layer at the interface was collected for subsequent staining.

Single-cell suspensions were first incubated with anti-mouse CD16/CD32 antibody (Thermo Fisher) for 10 minutes at 4°C to block Fc receptors. Cells were then stained with two separate antibody panels for 30 minutes at 4°C in the dark. Prior to sorting, cells were stained with eFluor™ 780 viability dye (Thermo Fisher) to exclude dead cells.

Panel 1: AF700 anti-mouse CD45 (Biolegend, 1:100), BV650 anti-mouse CD11b (Biolegend, 1:100), BV605 anti-mouse CD3 (Biolegend, 1:100), BV480 anti-mouse CD8 (Biolegend, 1:100), PE anti-mouse CD11c (Biolegend, 1:100), BV421 anti-mouse MHCII (Biolegend, 1:100), FITC anti-mouse NK1.1 (Biolegend, 1:100).

Panel 2: BV711 anti-mouse CD45 (Biolegend, 1:100), BV650 anti-mouse CD11b (Biolegend, 1:100), BV605 anti-mouse CD3 (Biolegend, 1:100), APC anti-mouse CD25 (Biolegend, 1:100), BV480 anti-mouse CD4 (Biolegend, 1:100).

Statistical analysis

Statistical analyses were conducted using GraphPad Prism software v10. The sample size is indicated in the figure legends, and details of statistical tests are provided in the respective figure legend where applicable. Appropriate tests, such as unpaired parametric Student’s t-test, ANOVA analysis, or unpaired non-parametric Mann-Whitney U test, were chosen based on the normality of data. Overall survival was estimated using the Kaplan-Meier method, and differences were assessed using the log-rank test. For infiltration and proximity analyses, two-way ANOVA followed by Šídák's multiple comparison test was performed to identify differences between two groups at different distances. A p-value less than 0.05 was considered statistically significant for all studies. *p<0.05, ** p<0.01, *** p<0.001, and **** p<0.0001; “n.s.” indicates not significant.

UK Biobank data

*Study population of UK Biobank*

We used the UK Biobank (https://www.ukbiobank.ac.uk/) is a comprehensive prospective cohort study, enrolled over 500,000 participants aged between 37 and 73 during the period of 2006 to 2010.^2^ Our aim was to compare the overall survival rates of pancreatic cancer patients with and without MASLD. We analyzed data from 3,943 pancreatic cancer patients, excluding those with pancreatic endocrine tumors, from the UK Biobank database. These patients were divided into two groups: those with severe MASLD (100 patients) and a control group (3,843 patients). Any individuals diagnosed with MASLD after their pancreatic cancer diagnosis were not included in the control group (n=82). This study was conducted under application number 69476. Survival curves were generated using Kaplan-Meier analysis which is suitable for estimating survival probabilities over time, and associations were assessed using Cox proportional hazards models to evaluate the effect of multiple covariates on survival outcomes. The UK Biobank received ethical approval from the Northwest–Haydock Research Ethics Committee (16/NW/0274). All participants signed informed consent forms, allowing their health records to be linked. **Supplementary Figure 1a** provides a visual representation of the study design. Exclusion criteria encompassed participants with a baseline diagnosis of pancreatic cancer and other tumors (n=43,218), hepatitis B/C and associated liver disease (n=2,128), missing data on key covariates such as BMI, smoking, and drinking status (n=6,087), and those diagnosed with pancreatic neuroendocrine tumors (n=57). Following these exclusions, the final analysis included 450,754 participants. During follow-up, 1,102 participants were lost, resulting in a total response rate of 99.8%.

*UK Biobank - Exposure and Outcome*

Severe MASLD was defined by International Classification of Diseases (ICD)-9 and ICD-10 codes, in line with previous studies.^3^ Specifically, MASLD was defined as ICD-9 5718 (other chronic nonalcoholic liver disease) using ICD-9, and as ICD-10 K76.0 (fatty [change of] liver, not elsewhere classified) and K75.8 (other specified inflammatory liver diseases) using ICD-10 and the latest Expert Panel Consensus Statement, MASLD was defined for the main analyses of this study.^4^ Notably, these participants exclude those with hepatitis b/c or alcohol-related liver disease or cirrhosis, which is defined by other ICD numbers. We obtained the date and cause of hospital admissions from inpatient records, which were linked to the Hospital Episode Statistics for England, Scottish Morbidity for Scotland, and the Patient Episode Database for Wales. **Supplementary Table 8** provides a more detailed explanation of the ICD codes used for identifying severe MASLD.

Incident PC cases were either identified using ICD-9 and ICD-10 codes from the Summary Diagnoses in hospital inpatient records or through self-reported cancer codes obtained during the Verbal Interview Medical Conditions at the Assessment Centre. To ensure that individuals with pancreatic endocrine tumors were not included, we used ICD-9 and ICD-10 codes from inpatient records and validated these exclusions through cancer tumor histology in the cancer registry, resulting in the exclusion of 57 individuals. **Supplementary Table 8** provides diagnostic codes used for PC and pancreatic endocrine tumors. **Supplementary Table 9** provides a detailed account of the variables with missing data, including their frequencies and proportions.

*UK Biobank - Covariates*

A directed acyclic graph that illustrates the relationships between the exposures, outcomes, and covariates was presented in Figure S1D. These variables encompassed demographic factors such as age, sex, race, alcohol consumption, Body Mass Index (BMI), smoking and drinking status, Index of Multiple Deprivation (IMD), blood pressure, low-density lipoprotein cholesterol (LDL-C), triglycerides, cholesterol, gamma glutamyl transferase, aspartate aminotransferase (AST), and alanine aminotransferase (ALT). Alcohol consumption and smoking status were categorized into three groups: never, ever, and current. Central obesity was determined based on the following criteria: for men, a waist circumference of ≥90 cm, a waist-to-hip ratio of ≥0.9, or a waist-to-height ratio of ≥0.5; for women, a waist circumference of ≥80 cm, a waist-to-hip ratio of ≥0.85, or a waist-to-height ratio of ≥0.5. Medical histories of hypertension, diabetes, and dyslipidemia were obtained through oral interviews conducted by trained nurses. These factors can impact MASLD and may confound the associations being studied.^3,5-8^ **Supplementary Table 10** provides detailed definitions of these medical conditions. Model adjustments were made in three stages. Model 1 incorporated basic demographics: sex, age, and the Index of Multiple Deprivation. Model 2 added lifestyle factors to Model 1, including alcohol intake, smoking status, and BMI. Model 3 further adjusted for triglycerides and histories of hypertension, dyslipidemia, and diabetes. Among the three models, Model 3, which had the lowest Akaike Information Criterion (AIC) and Bayesian Information Criterion (BIC) scores, indicating the best fit (**Supplementary Table 11**), was selected for the main analysis.

*UK Biobank - Statistical analysis*

After implementing an initial exclusion scheme, we addressed missing values in continuous variables through multiple imputation.^9^ We described baseline demographics and characteristics according to severe MASLD, presenting continuous variables as medians with quartile range [M (P25, P75)], and categorical variables as frequencies and percentages. We explored the associations of severe MASLD with PC using Cox-proportional hazard models. To mitigate potential immortal time bias,^10^ we treated MASLD as a binary time-varying variable, as done in previous studies.^11-13^ This means individuals were classified as unexposed until they were diagnosed with severe MASLD and were then assigned to the exposed group from the time of diagnosis until the end of follow-up.

We performed a variety of subgroup analyses, taking into account factors such as gender (male or female), age (<60 or ≥60), BMI (<24, 24–28, or ≥28), smoking status (never, former, or current), drinking status (never, former, or current), central obesity (yes or no), history of diabetes (yes or no), history of dyslipidemia (yes or no), and history of hypertension (yes or no). We used likelihood ratio tests to investigate interaction effects, comparing Cox models with and without an interaction term for each stratifying factor and exposure.

To ensure the reliability of our results, we carried out several sensitivity analyses: (1) to assess potential causal relationships, we excluded individuals diagnosed with PC within less than a year of exposure to severe MASLD; (2) we adjusted the Cox model to account for additional potential risk factors for PC and liver function biomarkers, including race, dyslipidemia, central obesity (other components of metabolic syndrome), and levels of AST, ALT, GGT, and LDL-C; (3) we excluded cases of severe MASLD that were present at baseline, considering only those diagnosed during the follow-up period; (4) individuals with missing data were also excluded.

All statistical analyses were conducted using R version 4.4.0 (R Foundation for Statis-tical Computing). We considered a two-sided P-value of less than 0.05 to be statistically significant.

Unique molecular identifiers (UID) RNA sequencing

8-week C57BL/6 male mice were fed with a normal diet or a CDAHFD for four weeks before being injected intrasplenically with 10^6^ KPC cells (n=3/group). Total RNA was extracted from metastatic liver tumor tissues using TRIzol in accordance with the manufacturer’s instructions. The quality of the RNA was assessed by analyzing the A260/A280 ratio using the NanodropTM OneC spectrophotometer (Thermo Fisher Scientific Inc). The integrity of the RNA was verified using 1.5% agarose gel electrophoresis, followed by the quantification of qualified RNA by Qubit3.0 with QubitTM RNA Broad Range Assay kit (Life Technologies, Q10210). A total of 2 micrograms of RNA were used for building a stranded RNA sequencing library using the KC-DigitalTM Stranded mRNA Library Prep Kit for Illumina® (DR08502, Wuhan Seqhealth Co., Ltd. China). The kit minimizes duplication bias in PCR and sequencing procedures by using a unique molecular identifier (UMI) consisting of 8 randomly generated bases to identify the pre-amplified cDNA molecules. The library products with base pair sizes ranging from 200 to 500 were subjected to enrichment, quantification, and sequencing using the Novaseq6000 sequencer (Illumina) with the PE150 model. Raw sequencing data was uploaded to the SRA database (PRJNA1372824) (<https://www.ncbi.nlm.nih.gov/sra/PRJNA1372824>)

Bulk RNA sequencing analysis

The sequencing data first underwent filtration using Trimmomatic (version 0.36), wherein low-quality reads were eliminated and runs containing adaptor sequences were trimmed. The Clean Reads underwent further processing using in-house scripts to remove any duplication bias that may have been introduced during library preparation and sequencing. Concisely, the clean reads were first classified into clusters based on their UMI sequences. The readings within the same cluster were subjected to pairwise alignment, and then, reads with a sequence similarity over 95% were isolated into a distinct sub-cluster. Once all sub-clusters were created, a multiple sequence alignment was conducted to get a single consensus sequence for each sub-cluster. Following these procedures, all mistakes and biases produced during PCR amplification or sequencing were eradicated. The UID RNA-seq experiment and high-throughput sequencing, as well as data processing and analysis, were performed by Seqhealth Technology Co., LTD (Wuhan, China). GSEA was performed using GSEA software (Broad Institute).

Single-cell RNA sequencing analysis

The raw and processed sequencing data have been deposited in NCBI’s Gene Expression Omnibus (GEO) under the accession number GEO: GSE125588 (mouse late KC [KIC] single cell RNA sequencing) and GEO: GSE166504 (mouse MASLD single cell RNA-sequencing). The gene-by-cell-expression matrix was loaded to the R package Seurat version 4.3.0 for downstream analysis.^14^ Low quality cells were filtered out based on mitochondrial gene content, number of genes detected. Data was scaled with ScaleData function and was subjected to dimensional reduction by principal component analysis. Cell clusters were identified via the Find Clusters function.

For GSE125588 data, filtered cells were clustered by using FindClusters function with resolution 0.8. Cell types were assigned based on the marker genes defined in the original study. 1022 cells from KIC group were included for subsequent analysis. For GSE166504 data, cell type labels were extracted directly from the well-annotated metadata from original study without performing independent clustering and 23709 cells from 30-week (NASH) group were included for subsequent analysis.

For integration analysis, each dataset was independently normalized using the SCTransform function to stabilize variance and mitigate technical artifacts. Prior to integration, 3,000 highly variable genes (HVGs) were selected across datasets using the SelectIntegrationFeatures function, followed by preprocessing with PrepSCTIntegration function. Datasets were then integrated using the FindIntegrationAnchors and IntegrateData functions, with the normalization.method parameter specified as 'SCT'. This anchor-based and sctransform-based integration workflow follows the official SeuratV4.3 vignette and effectively corrects batch effects, enabling robust downstream comparative analyses.

Differentially expressed genes (DEGs) between clusters were identified by using FindMarkers function with a Wilcoxon Rank Sum test. DEGs were defined as average log2 foldchange > 0.25 and p val adjust < 0.01. Cell communication analysis was performed by using the R package CellChat version 1.6.1.^15^ Cell communication with less than 10 cells was filtered out. The “create CellChat” function was used to create a CellChat object from integrated Seurat datasets. The “identify Over Expressed Genes”, “identify Over Expressed Interactions” functions were utilized to identify over-expressed ligands or receptors based on the Secreted Signaling subset from CellChat mouse database. The “compute Commun Prob” and “filter Communication” functions were used to computed communication probability and inferred cellular communication network. The “compute Commun Prob Pathway” and “aggregate Net” functions were ran to infer the cell-cell communication at a signaling pathway level between clusters.

Our selection of the late KIC (KIC) instead of the KPC mice for scRNA-seq analysis was driven by two key considerations: (1) Pathobiological relevance to early dissemination: As Stanger et al. experimentally demonstrated through lineage tracing, pancreatic cancer metastasis initiates during premalignant PanIN stages, with disseminated cells exhibiting prolonged dormancy before metastatic outgrowth.^16^ The earlier-stage KIC model thus better recapitulates this transitional biology of early dissemination—a mechanistic priority for our study investigating how fatty liver microenvironments attract pancreatic tumor cells. (2) In the KPC mouse pancreatic tumor sample, scRNA-seq captured approximately 1,000 cells, with fewer than 30 identifiable cancer cells (<3% of total). By contrast, the three late-stage KIC mouse samples collectively yielded ~3,000 cells, of which cancer cells constituted 13.73% (n≈412 cells). Thus, interrogating the KIC stage aligns with the clinically relevant window of early dissemination identified by Stanger et al.^16^

Histology

The tissues were fixed in a solution of 10% formalin buffered with PBS (AB1019, AOBO, China), embedded in paraffin, and cut into 4-5 µm slides. Briefly, the slides were deparaffinized with xylene and dehydrated in gradient ethanol, and the high-pressure antigen retrieval procedure was performed using the suitable antigen retrieval buffer as specified by the antibody manufacturer's instructions. The sections were treated with a 10% goat serum blocking solution (ZLI-9021; Beijing, China) for 30 minutes at 37℃, followed by incubation with corresponding primary antibodies overnight at 4℃, and an anti-rabbit/mouse secondary antibody (PV-9000; Beijing, China) 1 hour at room temperature. DAB (ZLI-9018; Beijing, China) staining was performed, followed by counterstaining with hematoxylin (G1140; Solarbio, Beijing, China). Aqueous mounting material was used to attach the slides after a distilled water wash. Images were captured on a microscope (Leica) and analyzed by ImageJ software. Expression levels were scored based on staining intensity (ImageJ). H&E slides were scanned (Zeiss) to calculate the number and size of the metastatic burden using the ZYFViewer software. The area occupied by metastatic foci was then divided by the total surface area.

Protein extraction and immunoblotting analysis

Mice liver metastatic issue samples were harvested and homogenized in a lysis buffer (P0013, Beyotime) using a Tissue Homogenizer Low-temperature (-40°C, KZ-III-FP, Servicebio). Protein lysis was separated using sodium dodecyl sulfate-polyacrylamide gel electrophoresis, transferred into a nitrocellulose membrane (Immobilon-P, Millipore), and then blocked with 5% BSA/TBST at room temperature for 1 h. The membranes were subjected to overnight incubation at 4 °C with primary antibodies. Following the washing step with TBST, the membranes were blotted using horseradish peroxidase-conjugated antibodies specific to either rabbit IgG or mouse IgG for a duration of 1 hour at room temperature. The signal was detected by the Pro e-BLOT Touch Imager using chemiluminescent substrate (WBKLS0500; Merck Millipore, Boston), and the band intensity was measured using ImageJ software (National Institutes of Health).

Proliferation, transmigration and invasion of PDAC cells

Cells were seeded in 96-well flat-bottom plates at a density of 5,000 cells (KPC and AsPC-1) per well and incubated with 100 µL of fresh DMEM containing 10% FBS medium with or without Mif (200ng/ml or 400ng/ml) were incubated together. Dynamically monitor cells for 36 hours via a live cell workstation.

To determine the optimal concentration of MIF for migration assays, KPC cells were seeded in Transwell (8 μm pores; BIOFIL, Guangzhou, China) inserts at a density of 5 × 10^4^ cells/well in the upper chamber. The lower chamber was treated with varying concentrations of MIF (0, 50, 100 and 200 ng/mL) along with 50 μg/mL (SDF-1) serving as a positive control. All cells were harvested after 36 h, resuspended in 100 μL 1% FBS medium, and seeded in the upper Transwell inserts migration chamber, with 600 μL 1% FBS in the lower chamber. Next, we evaluate the influence of MIF on the migratory behavior of KPC cells after transfection with si*Cd44* and si*Ctrl*. Briefly, following transfection with various siRNA formulations, cells were harvested and suspended in 1% FBS medium. Subsequently, 100 μL of cells (5 × 10^4^ cells) with different treatments were added to the upper chamber. The lower chamber was filled with 1% FBS medium containing various concentrations of MIF (0 and 200 ng/mL). After incubation for 36 hours at 37°C in a 5% CO2 atmosphere, KPC cells in the upper chamber were eliminated using cotton swabs. The invaded cells crossing the membrane into lower chamber were stained with 0.1% crystal violet at 37 °C for 40min. Three randomly selected areas at a magnification of 100 × of each sample using software, and the number of migratory cells was counted.

ASPC-1 cells were seeded in Transwell (8 μm pores; BIOFIL, Guangzhou, China) inserts at a density of 1 × 10^5^ cells/well in the upper chamber. The lower chamber was treated with varying concentrations of MIF (0, 50, 100, 200, 400 and 800 ng/mL) serving as a positive control. All cells were harvested after 36 h, resuspended in 100 μL 1% FBS medium, and seeded in the upper Transwell inserts migration chamber, with 600 μL 1% FBS in the lower chamber. After incubation for 36 hours at 37°C in a 5% CO2 atmosphere, ASPC-1 cells in the upper chamber were eliminated using cotton swabs. The invaded cells crossing the membrane into lower chamber were stained with 0.1% crystal violet at 37 °C for 40min. Three randomly selected areas at a magnification of 100 × of each sample using software, and the number of migratory cells was counted.

Cells were detached from the culture dish with trypsin and suspended in serum-free growth medium. Then, 100 μl of the cell suspension was transferred into 8-mm Transwell inserts, with 100 μl Matrigel coating. The Matrigel was diluted to a concentration of 250 μg/ml with serum-free medium. The lower chamber of the Transwell insert contains 600 μl of standard growth medium with or without Mif. After incubating for 36 h, cells were fixed with 3.7% formaldehyde and permeabilized with methanol, then stained with Wright-Giemsa stain. Cells remaining on the upper surface of the membranes were removed with a cotton swab. The invasive cells that had migrated to the bottom surface of the filters were quantified by counting the number in 12 microscopic fields per membrane at 10× magnification.

Characterization of EVs by Western Blot

EVs were resuspended in 20 µL of PBS were lysed in 50 µL of radioimmunoprecipitation assay (RIPA) buffer supplemented with a complete (Roche) protease inhibitor for 1 h at 4 °C. Then, the mixture was centrifuged at 12,000× g for 30 min at 4 °C, and the clarified lysate was transferred into a new 1.5 mL tube. Protein quantification was performed using the Pierce BCA Protein Assay kit (Thermo Fisher Scientific, Waltham, MA, USA) according to the manufacturer’s instructions. An equal amount of protein per sample was separated using SDS-PAGE on gradient (4–20%) and transferred to the membrane. Membranes were blocked in 5% nonfat milk in Tris-buffered saline with Tween 20 (TBS-T) buffer for a minimum of 1 h at room temperature, then probed (or re-probed) with the primary antibody overnight at 4 °C. Then, membranes were washed 4 times (10 min each), with 1× TBST (Tris-buffered saline, 0.1% Tween 20) by shaking gently and were then incubated with horseradish peroxidase conjugated-secondary antibody for 1 h at room temperature. Membranes were washed again 4 times with 1× TBST (10 min each), then treated with enhanced chemiluminescence kit-Pierce™ ECL Western Blotting Substrate (Thermo Scientific, Rockford, IL, USA) for 1 min.

SiRNA-mediated transient knockdown

The transient knockdown of Cd44 was performed using *Cd44* siRNA (si*Cd44*) (GenePharma, Shanghai, China), which is a pool of three target-specific nt siRNAs. KPC cells were plated in a 6-well plate at a concentration of 6×105/well in a volume of 2ml culture medium. On the following day, replace the original medium with 1 ml fresh medium, and then transfected with *Cd44* siRNA or non-targeting control siRNA (si*Ctrl*) at a concentration of 150 pmol/well using GP-transfect-Mate reagent (GenePharma, Shanghai, China). The medium was changed 24 h after transfection. After 48 h of infection, observe the expression of GFP with a fluorescence microscope, and lysates were collected for western blot verification.

Cell culture

Murine cell lines KPC was gifted from Professor Jing Xue (Shanghai Cancer Institute, Ren Ji Hospital, School of Medicine, Shanghai Jiao Tong University) and cultured under the recommended conditions following granted protocols, including the respective medium supplemented with 10% fetal bovine serum (FBS) and 1% antibiotics, and kept in 37 °C humidified incubators with 5% CO2. AML12 cell was supplied by the Cell Bank of Type Culture Collection of Chinese Academy of Sciences (Shanghai, China) and cultured in DMEM supplemented with 10% FBS, 1% antibiotics, 1% insulin–transferrin–selenium (Thermo Scientific), and 40ng/mL dexamethasone (Millipore, MA, USA) at 37 °C with 5% CO2 using a cell incubator. This study also used the human pancreatic ductal epithelial cell line AsPC-1. The cells were cultured in Roswell Park Memorial Institute (RPMI) 1640 medium supplemented with 10% fetal bovine serum, 1% insulin–transferrin–selenium, The cells were grown in a humidified atmosphere containing 5% CO2 at 37 °C.

Construction of the KPC-Luciferase cell line

Lentivirus production and transduction: The lentiviral transfer plasmid GV260 (Genechem, Shanghai, China), encoding the firefly luciferase reporter gene and a puromycin resistance gene (Puro), was used to generate lentiviral particles. Lentiviruses were produced in 293T cells using a second-generation packaging system (psPAX2 and pMD2.G plasmids). Briefly, 293T cells were seeded in 10-cm dishes at 5 × 10⁶ cells/dish and transfected using Lipofectamine 2000 (Invitrogen) with a mixture of 12 µg GV260, 9 µg psPAX2, and 3 µg pMD2.G. Viral supernatants were harvested 48 h and 72 h post-transfection, pooled, and filtered through a 0.45-µm membrane. Viral titers were determined using the Lenti-X™ Lentiviral Titer Kit (Takara).

Stable cell line selection: KPC cells were seeded in 6-well plates and transduced at ~70% confluence with lentiviral supernatant supplemented with 8 µg/mL Polybrene. After 24 h, the viral supernatant was replaced with fresh complete growth medium. Puromycin selection (50 µg/mL) was initiated 72 h post-transduction and maintained for 5–7 days, with the selective medium replenished every 2–3 days, until all control cells (non-transduced) died. This process established a stable KPC-Luciferase cell pool.

Luciferase reporter assay: Luciferase activity was quantified using a commercial assay kit (Promega). Cells were washed with PBS and lysed in 500 μL lysis buffer per well for 15 min at room temperature with gentle agitation. A 10-μL aliquot of lysate was transferred to a black 96-well plate, and luciferase activity was measured immediately after automated injection of the substrate solution. Readings were initiated after a 2-s delay followed by a 10-s integration period to confirm stable reporter gene expression.

Supplementary Text

Results – UK Biobank

Characteristics of the study population are detailed in **Supplementary Table 1**. Compared to those without MASLD, individuals with MASLD were more likely to be female, have lower income, and possess a history of smoking, hypertension, dyslipidemia, diabetes, and central obesity. Furthermore, those diagnosed with severe MASLD exhibited higher waist and hip circumferences, BMI, and triglyceride levels. This data underscores the significant differences in health and demographic profiles between individuals with and without MASLD.

Subgroup analyses were conducted based on age, sex, BMI, central obesity, smoking and drinking status, and histories of diabetes, hypertension, and dyslipidemia (**Supplementary Fig. 6**). The increased risk of PDAC associated with MASLD was consistently observed across subgroups defined by age, sex, BMI, central obesity, smoking, and drinking. No significant interaction was found between MASLD and age, sex, BMI, smoking, or drinking. However, central obesity significantly modified the effect of MASLD on PDAC risk (P for interaction = 0.037). In terms of medical history, MASLD was a significant risk factor for PDAC development in individuals without diabetes (HR: 3.72; 95% CI: 2.83-4.90). Conversely, the association between MASLD and PDAC in diabetic individuals was not statistically significant (HR: 2.14; 95% CI: 0.99-4.66), with no significant interaction between diabetes and MASLD (P for interaction = 0.25). Furthermore, dyslipidemia and hypertension significantly attenuated the effect of MASLD on PDAC risk. The adjusted HR was 1.02 (95% CI: 0.38-2.77) in individuals with dyslipidemia com-pared to 4.01 (95% CI: 3.07-5.23) in those without dyslipidemia (P for interaction = 0.012). For hypertension, the HR was 1.73 (95% CI: 0.94-3.17) compared to 4.37 (95% CI: 3.29-5.81) in those without hypertension (P for interaction = 0.007). These findings underscore the complex interplay between MASLD and various demographic and clinical factors in influencing PDAC risk.

To validate the strength of our results, we performed multiple sensitivity analyses (**Supplementary Tables 12-15**). The link between severe MASLD and PDAC was consistent with our primary results, even after excluding participants with less than a year’s exposure to severe MASLD who were diagnosed with PDAC (HR: 1.62; 95% CI: 1.11-2.35), as shown in **Supplementary Table 12**. Incorporating additional variables into the model did not change the relationship between MASLD exposure and PDAC (HR: 3.33; 95% CI: 2.56-4.32) (**Supplementary Table 13**). The relationship between MASLD exposure and PDAC remained similar to our main findings even after excluding participants with MASLD at the start of the study (HR: 4.48; 95% CI: 3.46-5.80) (**Supplementary Table 14**). After the initial process of inclusion and exclusion, and further removal of individuals with missing data, the link between severe MASLD and PC stayed consistent with our primary results (HR: 2.86; 95% CI: 2.18-3.75) (**Supplementary Table 15**).

Discussion – UK Biobank

Despite several strengths in our study, it is necessary to acknowledge the following limitations. (1) While we eliminated the influence of pancreatic endocrine tumors, we could not rule out the impact of other malignant pancreatic tumor types (such as pancreatic acinar cell carcinoma, cystadenocarcinoma, etc.). However, PDAC represents 90% of pancreatic cancers, with pancreatic endocrine tumors and other types accounting for 5% and a small proportion, respectively; (2) The study’s generalizability is limited due to the population being predominantly older adults (average age 57) and white individuals (90.47%). However, as 90% of newly diagnosed pancreatic cancer patients are over 55 years old,^17^ this cohort ensures a sufficient number of new PDAC participants in the follow-up; (3) The participant inclusion criteria of UK Biobank are based on NAFLD instead of MASLD, and we excluded those with ALD according to the ICD codes. However, we cannot completely rule out the synergy between alcohol-associated and MASLD.

**
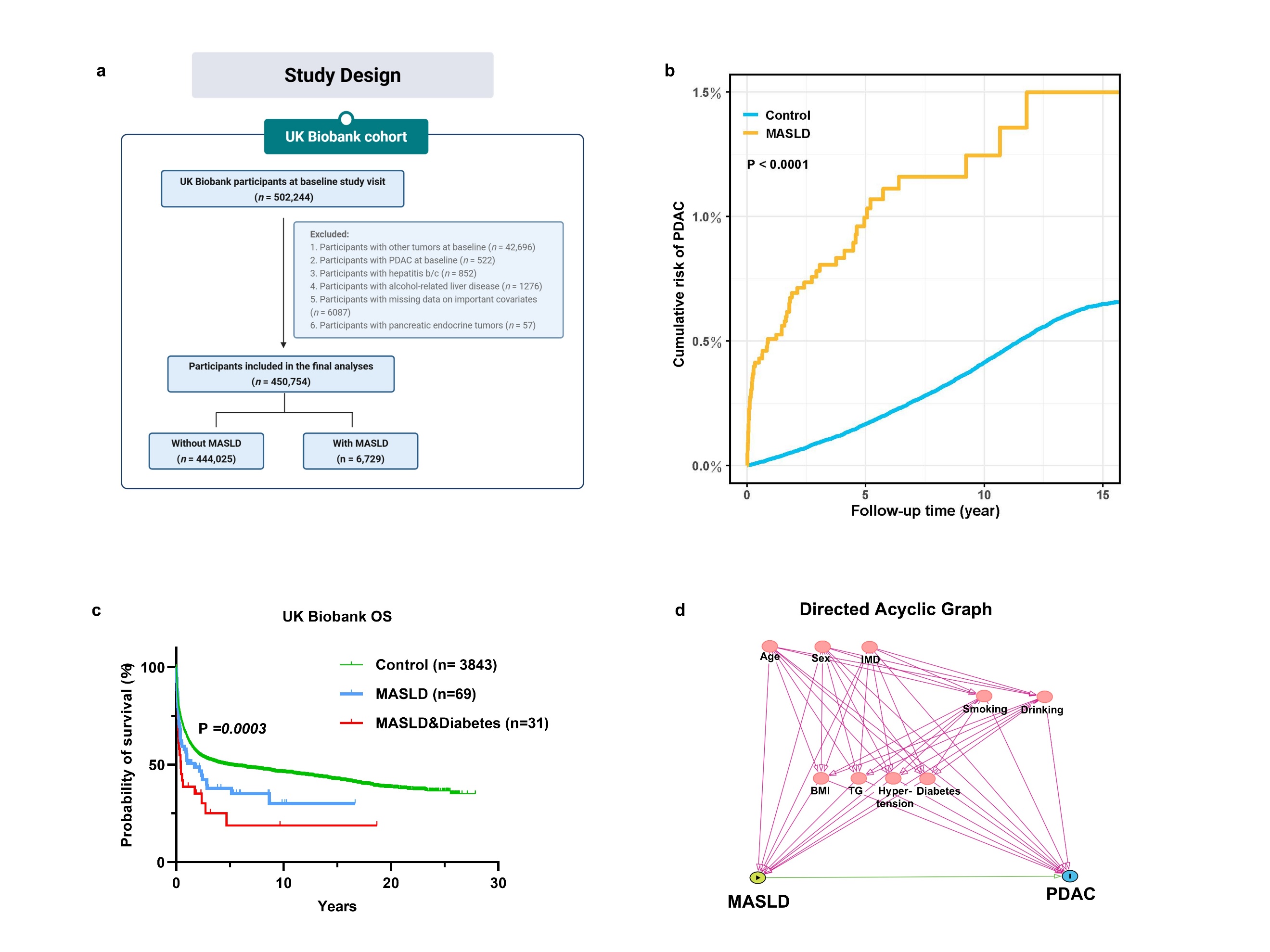
**

Figure. S1. (related to Figure 1). MASLD patients are associated with an increased risk of PDAC in UK Biobank. (a) Flowchart showing the criteria of the study cohort from participants in the UK Biobank. (b) Kaplan-Meier estimates of cumulative risk of PC with or without MASLD exposure. (c) Kaplan-Meier survival analysis. Survival curves are shown for the control group, the MASLD group, and the MASLD with diabetes group. Differences between groups were assessed using the log-rank test. (d) Directed acyclic graph (DAG) explaining the association between the exposures, the outcome, and covariates included in the analyses. DAG was drawn using <http://www.dagitty.net/>. IMD: Index of multiple deprivation, TG: triglyceride. PDAC, pancreatic duatal adenocarcinoma. MASLD, Metabolic Dysfunction Associated Steatotic Liver Disease.


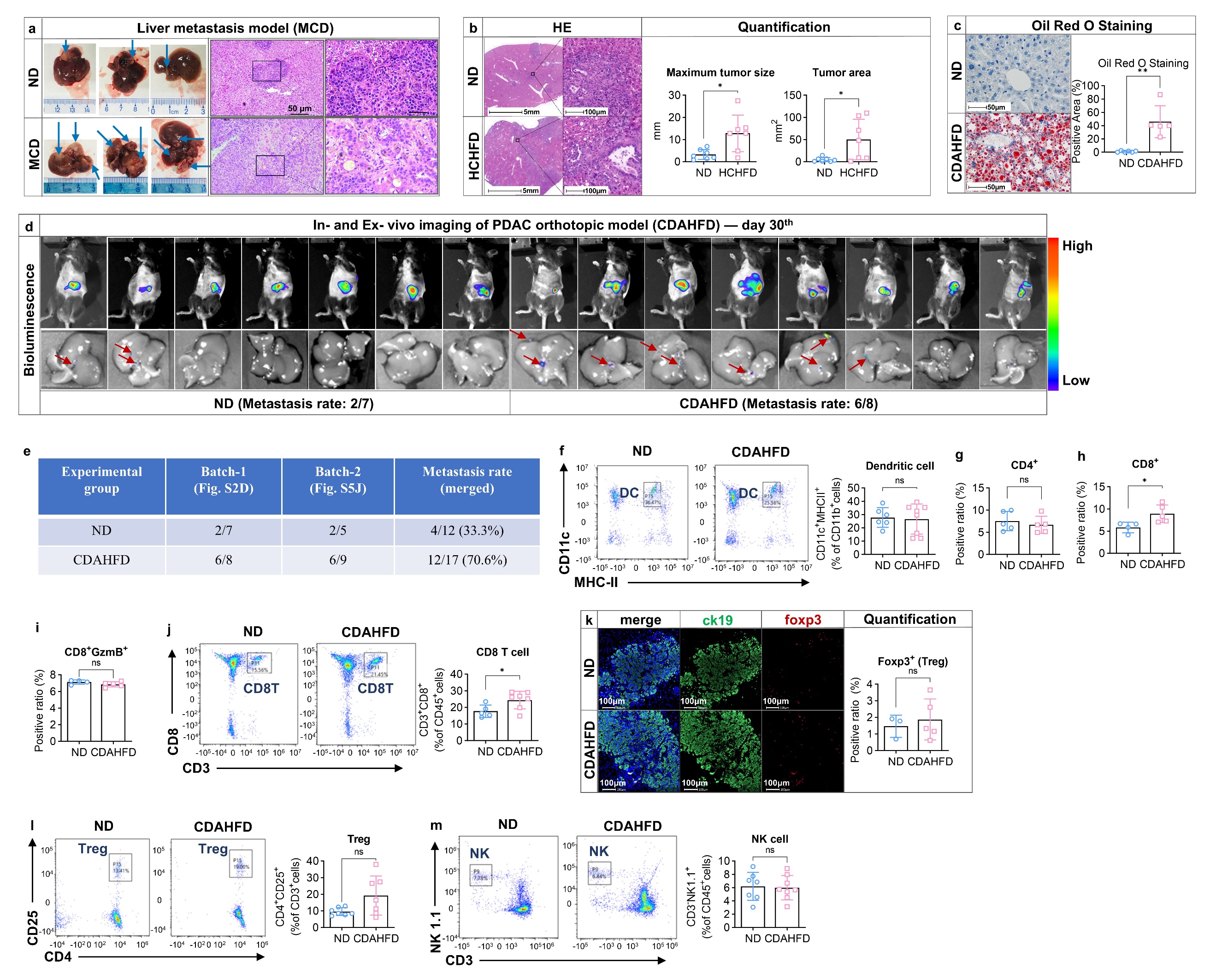


Figure. S2. (related to Figure 2). MASLD enhances metastatic tumor growth in the liver. (a) After 4 weeks of MCD induction, mice were injected with KPC cells in the spleen and sacrificed on the 15th day (n=7). Representative macroscopic appearance of the liver metastases and HE staining. (b) After 4 weeks of CDAHFD induction, mice were injected with KPC cells in the spleen and sacrificed on the 15^th^ day (n=7). (c) Representative oil red staining and quantification of the area of Oil red in liver tissues (n=5-6). (d) After 4 weeks of CDAHFD feeding, mice were injected with 2.5×10^5^ KPC-luciferase cells in the pancreas. Bioluminescence and ex vivo fluorescence of liver 4 weeks after pancreas injection. *Ex vivo*fluorescent images of harvested livers from both ND- and CDAHFD-fed mice and the metastasis rates were counted based on bioluminescence (n=7-8). (e) Combined metastatic rates derived from the control diet and CDAHFD groups across two experimental batches of orthotopic pancreatic models presented in Fig. S2d (batch-1: ND group, n=7; CDAHFD group, n=8) and Fig. S5j (batch-2: ND group, n=5; CDAHFD: batch-2, n=9). (f-k) After 4 weeks of CDAHFD feeding, mice were injected with 10^6^ KPC cells in the spleen and sacrificed on the 15^th^ day. CD45^+^ immune cells were enriched from single cell suspension of liver metastases in ND- and CDAHFD-fed mice. Dendritic cells (CD11c^+^MHC-II^+^) (f), CD8 T cells (CD3^+^CD8^+^) (j), Tregs (CD4^+^CD25^+^) (l), NK cells (CD3^-^NK1.1^+^) (m) were analyzed by flow cytometry (n = 5-8/group). (g-i) The quantification of CD4, CD8 and GmzB were examined mIHC and quantified by Halo software using HighPlex FL v4.2.14 module (n=4-5). (k) The expression of FOXP3 was examined by mIHC, and the ratio of FOXP3^+^ cells in the tumor area was quantified by Halo software using HighPlex FL v4.2.14 module (n=3-5). MCD, methionine- and choline-deficient diet; DC, dendritic cell; NK, natural killer cell.


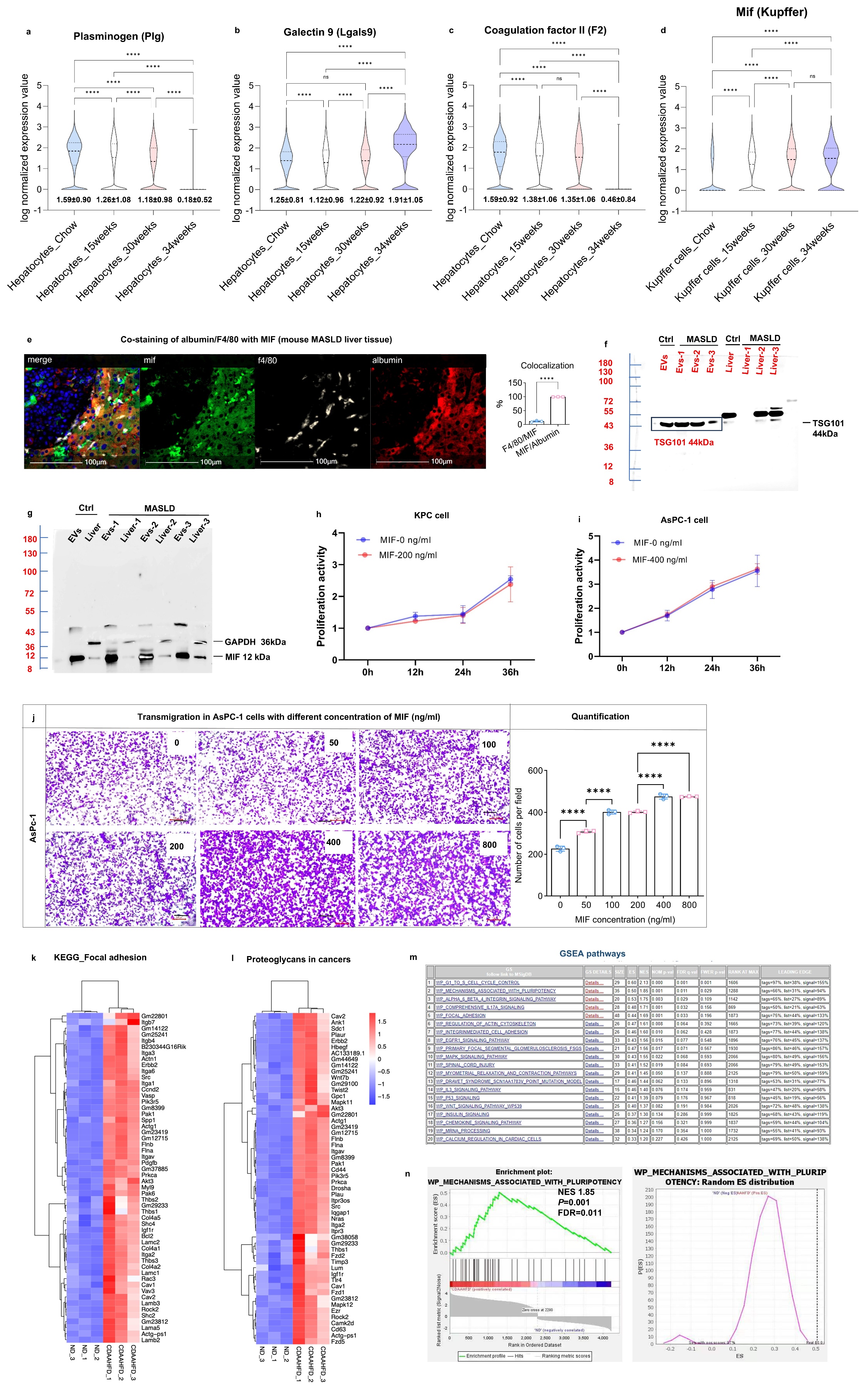


Figure. S3. (related to Figure 3). Liver metastasis progression involves the MIF-CD44 axis triggered by MASLD. (a-c) Quantification of RNA expressions of *Plg*, *Lgals9* and *F2* in the hepatocytes with different duration of high fat feeding based on GSE166504. (d) Quantification of *Mif* RNA expression in the Kupffer cells with different duration of high fat feeding based on GSE166504. (e) Co-localization of MIF, F4/80 and albumin (marker for hepatocytes) were examined by mIHC, and the co-localization ratios of MIF^+^F4/80^+^ and MIF^+^albumin^+^ cells were quantified by Halo software using HighPlex FL v4.2.14 module. (f-g) EVs and cell lysates extracted from the livers of the Ctrl (n=1) and MASLD mice (n=3) were used for Western blotting for EV’s marker, TSG101, MIF and GAPDH. (h-i) Proliferation activities of KPC and AsPC-1 cells with stimulation of MIF protein (n=3). (j) MIF protein with different concentrations (0, 50, 100, 200, 400, 800 ng/ml) was added in the lower chambers of 24-well transwell plates and 1 × 10^5^ AsPC-1 cells were seeded in the upper chambers. After 36 hours of incubation, we performed staining of the invaded cells on the lower membrane surface with 0.1% crystal violet and counted their numbers (n=3). (k-l) Heatmap of the top three upregulated KEGG pathways (Focal adhesion and Proteoglycans) based on transcriptomic analysis of liver metastatic tissues (n=3 per group) (CDAHFD model). (m) Top 20 GSEA pathways involved in the MASLD-induced metastasis based on transcriptomic analysis of liver metastatic tissues (n=3 per group) (CDAHFD model). (n) GSEA for gene sets associated with tumor pluripotency gene sets in metastatic liver tissues from ND-fed and CDAHFD-fed mice. NES, normalized enrichment score; FDR, false discovery rate. EVs, extracellular vesicles. Scale bar was indicated in the individual figures. Representative pictures are shown. Data are shown as mean ± SD per group. Unpaired parametric Student’s t-test or one-way ANOVA was performed to identify differences between two groups or four groups, respectively. A *p*-value less than 0.05 was considered statistically significant. **p*<0.05, ** *p*<0.01, *** *p*<0.001, and **** *p*<0.0001; “n.s.” indicates not significant.


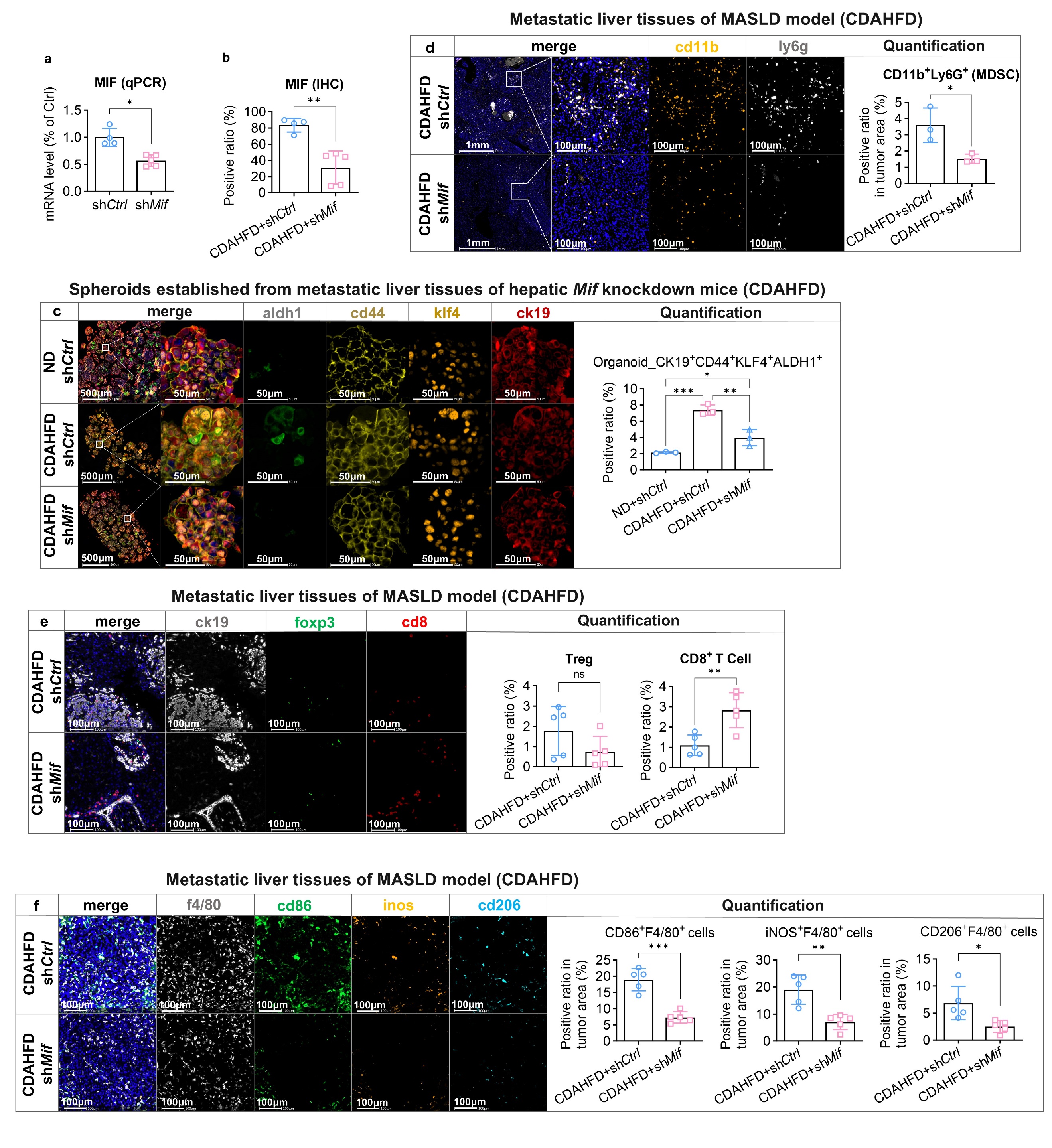
Figure. S4. (related to Figure 4). CD44 is the critical factor for MIF-mediated liver metastasis in MASLD. (a) After 4 weeks of CDAHFD induction, AAV-sh*Gfp* or AAV_sh*Mif* particles were injected through the tail vein to achieve hepatic MIF knockdown. Liver tissues were harvested for RNA extraction after 4 weeks of virus injection and examined. Hepatic mRNA levels of *Mif* were examined by qPCR (n=4-5/group). (b) MIF expression was examined by mIHC and quantified by Halo software corresponding to Figure 4g. (c) Sphere formation assay was performed on metastatic tissues from Ctrl+sh*Ctrl*, CDAHFD+sh*Ctrl*, and CDAHFD+sh*Mif* group, respectively. 3000/well were seeded in 96-well ultra-low attachment plates in stem cell medium. 7 days later, the spheroids were resuspended and seeded again (n=3). A 4-color mIHC staining including ALDH1, CD44, KLF4, and CK19 was performed on spheroids collected after 14 days of cultivation. Representative images including whole slide, merged channels and separate channels were shown. The total number of ALDH1^+^CD44^+^KLF4^+^CK19^+^ cells in the tumor area were quantified by Halo software using HighPlex FL v4.2.14 module in the column chart. (d) The expressions of CD11b and Ly6G were examined by mIHC, and the ratio of CD11b^+^Ly6G^+^ cells in the tumor area was quantified by Halo software using HighPlex FL v4.2.14 module (n=3). (e) The expressions of FOXP3 and CD8 were examined by mIHC, and the ratio of FOXP3^+^ and CD8^+^ cells in the tumor area was quantified by Halo software using HighPlex FL v4.2.14 module (n=5). (f) The total number of CD206^+^F4/80^+^, CD86^+^F4/80^+^ and iNOS^+^/F4/80^+^ cells were quantified in tumor areas by Halo software using HighPlex FL v4.2.14 module in the column chart (n=5). Data are shown as mean ± SD per group. Unpaired parametric Student’s t-test or one-way ANOVA was performed to identify differences between two groups or among different groups, respectively. A p-value less than 0.05 was considered statistically significant. *p<0.05, ** p<0.01, *** p<0.001, and **** p<0.0001.


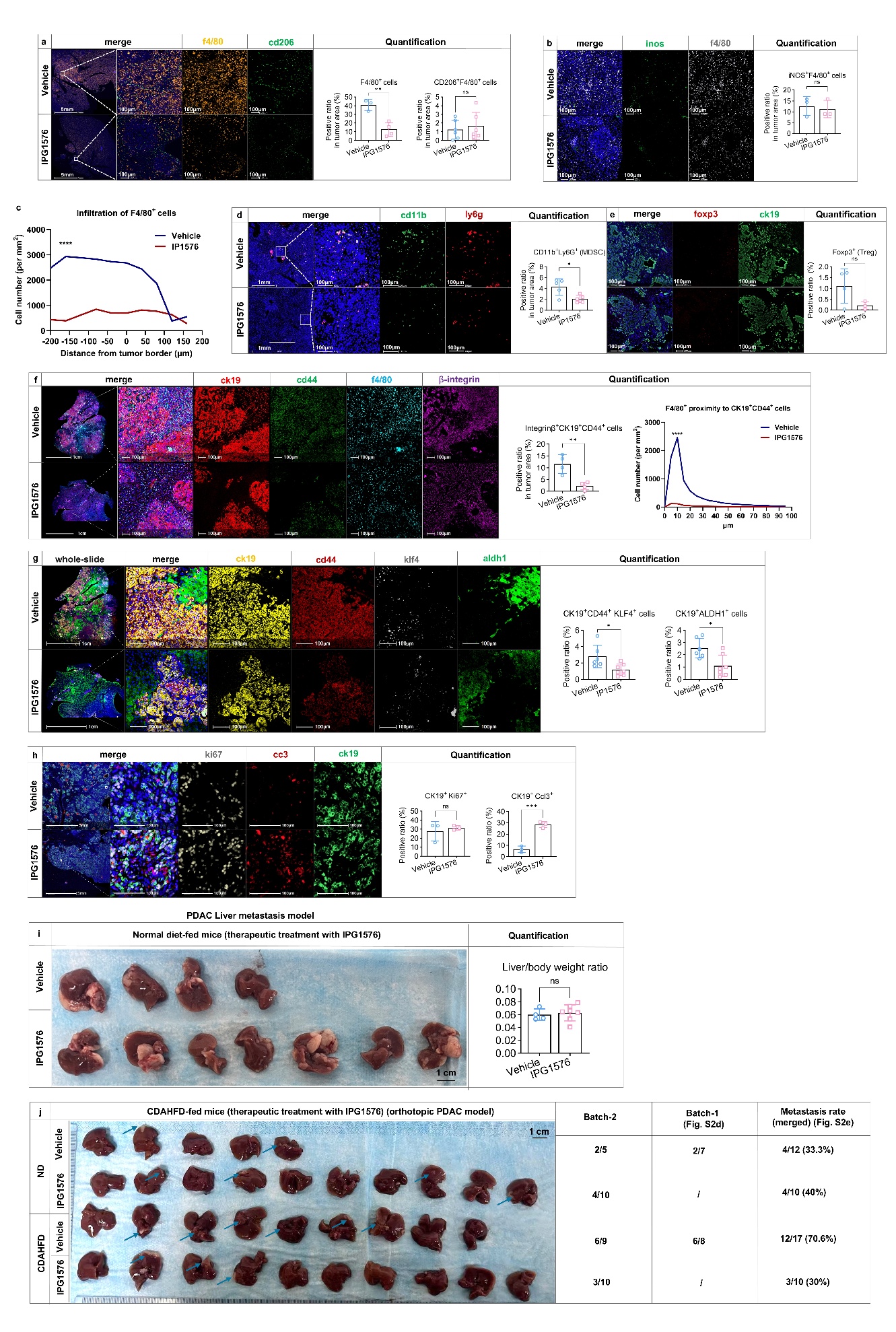


Figure. S5. (related to Figure 5). Pretreatment of MIF inhibitor or therapeutic targeting of MIF-CD44 axis inhibits metastatic tumor growth in orthotopic and metastatic models. Mice were fed with a CDAHFD for 4 weeks before the establishment of the liver metastasis model. The compound IPG1576 (30mg/kg/d) was administered twice daily via oral gavage and commenced seven days prior to the establishment of the metastasis model and continued for a duration of 14 days. An equivalent quantity of solution, 5%DMSO and 95% (20% [2-Hydroxypropyl]-β-cyclodextrin), was used as vehicle for IPG1576 (n=6-7) (A-H). Different panels of mIHC staining were performed on metastatic liver tissues from vehicle and IPG1576 group (pretreatment), respectively. Representative images including merged channels and separate channels were shown. (a-b) The total number of CD206^+^F4/80^+^ and iNOS^+^/F4/80^+^ cells were quantified in tumor areas by Halo software using HighPlex FL v4.2.14 module in the column chart (n=3-6). (c) The number of F4/80^+^ cells per mm^2^ tissue across the tumor border was quantified based on the infiltration rainbow. (d) The expressions of CD11b and Ly6G were examined by mIHC, and the ratio of CD11b^+^Ly6G^+^ cells in the tumor area was quantified by Halo software using HighPlex FL v4.2.14 module (n=5). (e) The expressions of FOXP3 was examined mIHC, and the ratio of FOXP3^+^ cells in the tumor area was quantified by Halo software using HighPlex FL v4.2.14 module (n=3-4). (f) A 4-color mIHC staining including CK19, CD44, F4/80, and integrinβ was performed on metastatic liver tissues from vehicle and IPG1576 group, respectively. The total number of integrinβ^+^CD44^+^ cancer cells were quantified by Halo software using HighPlex FL v4.2.14 module (n=4). Proximity analysis was performed on CD44^+^CK19^+^ cells and F4/80^+^ cells using Halo software. (g) A 4-color mIHC staining including CK19, CD44, KLF4 and ALDH1 was performed on metastatic liver tissues from vehicle and IPG1576 group, respectively. The quantification of stem-like cancer cells which are positive for CD44, ALDH, or CD44/KLF4 by Halo software using HighPlex FL v4.2.14 module (n=6-7). (h) Proliferative and apoptotic cancer cells were examined by mIHC staining of a 3-color mIHC staining including CK19, Ki67 and Cc3 on metastatic liver tissues from vehicle and IPG1576 group, respectively. The ratio of CK19^+^ Ki67^+^ cells and CK19^+^ Cc3^+^ cells were quantified by Halo software using HighPlex FL v4.2.14 module (n=3). (i) Mice were fed with a normal diet before the establishment of the liver metastasis model. Five days after intrasplenic injection of KPC cells (5×10^5^), IPG1576 (30mg/kg/d) was administered twice daily for a duration of 14 days (n=4-7). Representative macroscopic appearance of the liver metastases and the liver to body weight ratio were shown. (j) Mice were fed with a normal diet or a CDAHFD for four weeks before the establishment of the liver metastasis model. Five days after orthotopic injection of KPC cells (2.5×10^5^), IPG1576 (30mg/kg/d) was administered twice daily for a duration of 14 days (n=5-10). Representative macroscopic appearance of the liver metastases and the ratio of hepatic metastases were recorded. Combined metastatic rates derived from the ND and CDAHFD groups across two experimental batches of orthotopic pancreatic models presented in Fig. S2d (batch-1: ND group, n=7; CDAHFD group, n=8) and Fig. S5j (batch-2: ND group, n=5; CDAHFD: batch-2, n=9). Scale bar was indicated in the individual figures. Representative pictures are shown. Data are shown as mean ± SD per group. Unpaired parametric Student’s t-test was performed to identify differences between two groups. For proximity analyses, two-way ANOVA followed by Šídák's multiple comparison test was performed to identify differences between two groups at different distances. A p-value less than 0.05 was considered statistically significant. *p<0.05, ** p<0.01, *** p<0.001, and **** p<0.0001; “n.s.” indicates not significant. cc3, Cleaved caspase-3.


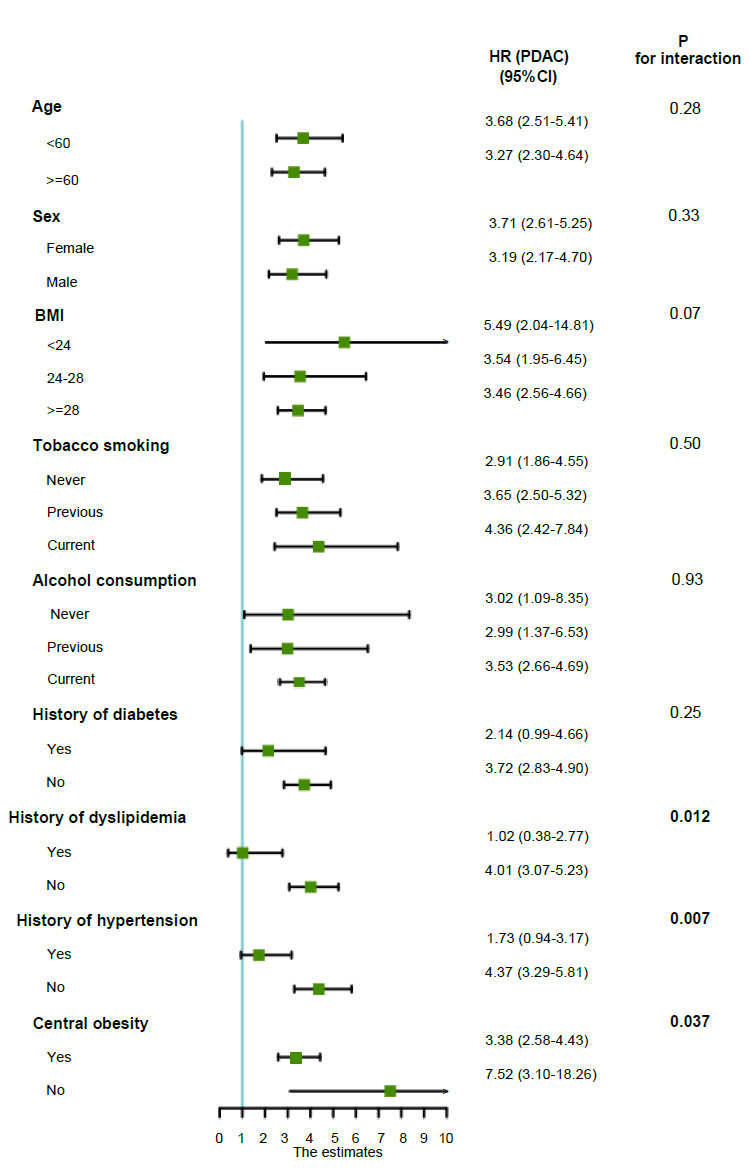


Figure. S6. Subgroup analysis of the association between MASLD and PDAC. Data presented as hazard ratio (95% CI), adjusted with model 3 for age, sex, BMI, IMD, TG, smoking and drinking status, hypertension, and diabetes. TG: triglyceride; BMI: body mass index; IMD: Index of Multiple Deprivation; CI: confidence interval.

Table S1.

Baseline characteristics of participants grouped by MASLD in the UK biobank

|  | **ALL** | **Control** | **MASLD** |
| --- | --- | --- | --- |
|  | **N=450754** | **N=444025** | **N= 6729** |
| Follow-up time (year) | 14.50 [13.76, 15.22] | 14.50 [13.76, 15.22] | 14.45 [13.58, 15.12] |
| Age (year) | 57.00 [50.00, 63.00] | 57.00 [50.00, 63.00] | 58.00 [51.00, 63.00] |
| Sex（Male） | 208907 (46.30) | 205673 (46.30) | 3234 (48.10) |
| Ethnicity (White) | 407783 (90.50) | 401768 (90.50) | 6015 (89.40) |
| BMI（kg/m^2^） | 26.74 [24.14, 29.90] | 26.69 [24.11, 29.82] | 30.55 [27.49, 34.41] |
| Waist circumference (cm) | 90.00 [80.00, 99.00] | 90.00 [80.00, 99.00] | 100.00 [91.00, 109.00] |
| Hip circumference (cm) | 102.00 [97.00, 108.00] | 102.00 [97.00, 108.00] | 107.00 [101.60, 115.00] |
| Diastolic pressure (mmHg) | 82.00 [75.00, 89.00] | 82.00 [75.00, 89.00] | 84.00 [77.00, 91.00] |
| Systolic pressure (mmHg) | 138.00 [126.00, 152.00] | 138.00 [126.00, 152.00] | 141.00 [129.00, 154.00] |
| IMD | 12.66 [7.08, 23.14] | 12.62 [7.07, 23.02] | 17.06 [9.27, 30.77] |
| ALT (U/L) | 20.16 [15.43, 27.42] | 20.08 [15.39, 27.25] | 28.21 [19.76, 41.33] |
| AST (U/L) | 24.30 [21.00, 28.80] | 24.30 [21.00, 28.70] | 27.70 [22.70, 35.70] |
| Cholesterol (mmol/L) | 5.65 [4.91, 6.42] | 5.65 [4.91, 6.42] | 5.43 [4.61, 6.30] |
| GGT (U/L) | 26.20 [18.50, 40.70] | 26.10 [18.40, 40.40] | 41.90 [26.70, 72.20] |
| LDL_C (mmol/L) | 3.52 [2.95, 4.11] | 3.52 [2.95, 4.12] | 3.41 [2.79, 4.06] |
| TG (mg/dL) | 131.00 [92.29, 189.81] | 130.46 [91.94, 188.92] | 171.83 [120.46, 244.72] |
| UA (μmol/L) | 303.10 [250.50, 360.90] | 302.50 [250.10, 360.30] | 336.80 [283.10, 395.50] |
| Tobacco smoking, n (%) |  |  |  |
| Never | 249261 (55.30) | 246135 (55.40) | 3126 (46.50) |
| Previous | 154320 (34.20) | 151659 (34.20) | 2661 (39.50) |
| Current | 47173 (10.50) | 46231 (10.40) | 942 (14.00) |
| Alcohol status, n (%) |  |  |  |
| Never | 19928 (4.40) | 19472 (4.40) | 456 (6.80) |
| Previous | 15538 (3.40) | 15085 (3.40) | 453 (6.70) |
| Current | 415288 (92.10) | 409468 (92.20) | 5820 (86.50) |
| Diabetes | 9073 (2.00) | 8523 (1.90) | 550 (8.20) |
| Dyslipidemia | 14588 (3.20) | 13943 (3.10) | 645 (9.60) |
| Hypertension | 32544 (7.20) | 31281 (7.00) | 1263 (18.80) |
| Central obesity | 330393 (73.30) | 324056 (73.00) | 6337 (94.20) |
| PDAC | 2804 (0.60) | 2678 (0.60) | 126 (1.90) |

Continuous values were presented as median (interquartile range) and categorical variables were presented as counts (percentages); BMI: body mass index; IMD: Index of Multiple Deprivation; TG: triglycerides; LDL_ C: low density lipoprotein; GGT: gamma-glutamyl transferase; AST: aspartic transaminase; ALT: alanine aminotransferase; UA: uric acid; PDAC: pancreatic ductal adenocarcinoma; MASLD: metabolic dysfunction-associated steatotic liver disease.

Table S2.

PDAC incidence grouped by MASLD^a^

|  | Not exposed to MASLD | Exposed to MASLD |
| --- | --- | --- |
| Person-years at risk | 6,340,792 | 35,183 |
| Incident cases | 2,743 | 61 |
| Incident cases per 100,000 | 43 | 173 |
| person-years |  |  |
| ^a^For those diagnosed with severe MASLD during the follow-up, their person-years at risk from the baseline to the diagnosis of severe MASLD were assigned to the “not exposed to severe MASLD” group, while their person-years at risk since the diagnosis of severe MASLD were assigned to the “expose to severe MASLD” group. | | |

Table S3.

Demographic and clinical characteristics of PDAC patients with or without MASLD - BJ cohort (n = 661)

| Variables | Total  (n = 661) | | Control  (n = 491) | MASLD  (n = 170) | *P* |
| --- | --- | --- | --- | --- | --- |
|  |  |  |  |  |  |
| Age (year) | 62.00 (55.00, 68.00) | | 63.00 (56.00, 69.00) | 59.50 (53.00, 66.00) | **0.0006** |
| Leukocyte (10^9^/L) | 5.88 (4.86, 7.04) | | 5.73 (4.81, 6.86) | 6.30 (5.17, 7.53) | **0.0008** |
| Neutrophile Granulocyte (10^9^/L) | 3.76 (2.92, 4.64) | | 3.71 (2.86, 4.56) | 4.13 (3.13, 5.08) | **0.0009** |
| Lymphocyte (mg/L) | 1.47 (1.11, 1.88) | | 1.47 (1.13, 1.87) | 1.49 (1.07, 1.90) | 0.8304 |
| ALT (U/L) | 26.00 (15.00, 154.00) | | 22.00 (13.00, 122.50) | 38.50 (20.25, 266.00) | **<.0001** |
| AST (U/L) | 23.00 (18.00, 95.00) | | 22.00 (17.00, 70.50) | 28.00 (20.00, 139.00) | **0.0005** |
| ALT/AST | 1.05 (0.75, 1.46) | | 1.00 (0.72, 1.38) | 1.21 (0.94, 1.69) | **<.0001** |
| TBIL (μmol /L) | 15.00 (10.30, 80.40) | | 13.80 (9.70, 44.90) | 19.80 (11.83, 160.48) | **<.0001** |
| DBIL (μmol/ L) | 5.20 (3.40, 60.60) | | 4.70 (3.20, 32.70) | 7.40 (3.90, 119.60) | **<.0001** |
| GGT (U/L) | 37.00 (19.00, 360.00) | | 32.00 (18.00, 291.50) | 76.00 (25.00, 658.25) | **<.0001** |
| LDH (IU/L) | 186.00 (162.00, 219.00) | | 184.00 (161.00, 212.50) | 189.50 (163.50, 246.00) | **0.0108** |
| ALP (IU/L) | 102.00 (75.00, 297.00) | | 94.00 (72.00, 274.50) | 142.50 (83.25, 350.25) | **0.0007** |
| UA (μmol/L) | 266.00 (215.00, 325.00) | | 264.00 (214.50, 323.50) | 272.00 (221.50, 326.00) | 0.4594 |
| BUN (mg/dL) | 4.62 (3.74, 5.64) | | 4.65 (3.79, 5.70) | 4.51 (3.70, 5.40) | 0.1275 |
| Cr (μmol/L) | 62.00 (53.00, 72.00) | | 61.00 (53.00, 72.00) | 66.00 (55.00, 76.00) | **0.0287** |
| Alb (g/L) | 43.00 (40.00, 45.00) | | 42.00 (40.00, 45.00) | 43.00 (40.00, 46.00) | 0.1110 |
| CA199 (U/ml) | 177.00 (57.20, 552.20) | | 174.00 (60.05, 560.30) | 183.15 (53.08, 526.25) | 0.8047 |
| ACE (ng/ml) | 3.22 (2.01, 5.36) | | 3.20 (2.00, 5.30) | 3.43 (2.10, 5.77) | 0.3228 |
| Gender, n (%) |  | |  |  | **0.0008** |
| Male | 312 (47.20) | | 213 (43.38) | 99 (58.24) |  |
| Female | 349 (52.80) | | 278 (56.62) | 71 (41.76) |  |
| Diabetes, n (%) |  | |  |  | 0.4189 |
| No | 486 (73.52) | | 357 (72.71) | 129 (75.88) |  |
| Yes | 175 (26.48) | | 134 (27.29) | 41 (24.12) |  |
| Smoke, n (%) |  | |  |  | 0.0596 |
| No | 509 (77.00) | | 387 (78.82) | 122 (71.76) |  |
| Yes | | 152 (23.00) | 104 (21.18) | 48 (28.24) |  |
| Radical operation, n (%) | |  |  |  | 0.3333 |
| No | | 244 (36.91) | 176 (35.85) | 68 (40.00) |  |
| Yes | | 417 (63.09) | 315 (64.15) | 102 (60.00) |  |
| Hepatic metastases, n (%)  No  Yes | | 464 (72.47)  197 (27.53) | 400 (83.50)  91 (16.50) | 64 (40.59)  106 (59.41) | **<.0001** |
| Lung metastasis, n (%) | |  |  |  | 0.0687 |
| No | | 575 (86.99) | 434 (88.39) | 141 (82.94) |  |
| Yes | | 86 (13.01) | 57 (11.61) | 29 (17.06) |  |
| Peritoneal metastasis, n (%) | |  |  |  | 0.4174 |
| No | 572 (86.54) | | 428 (87.17) | 144 (84.71) |  |
| Yes | 89 (13.46) | | 63 (12.83) | 26 (15.29) |  |

Continuous values were presented as median (interquartile range) and categorical variables were presented as counts (percentages); ALT: alanine aminotransferase; AST: aspartic transaminase; TBIL: total bilirubin; DBIL: direct bilirubin; LDH: lactic dehydrogenase; GGT: gamma-glutamyl transferase; ALP: alkaline phosphatase; UA: uric acid; BUN: blood urea nitrogen; Cr: creatinine; Alb: albumin; CEA: carcinoembryonic antigen.

**ZJ cohort**

| Variables | Total  (n = 276) | | Control  (n = 242) | | MASLD  (n = 34) | *P* |
| --- | --- | --- | --- | --- | --- | --- |
|  |  |  |  |  |  |  |
| Age (year) | | 64.50 (59.00, 70.25) | | 65.00 (59.00, 71.00) | 60.00 (51.50, 65.75) | **0.0010** |
| ALB (g/L) | | 43.00 (39.40, 45.02) | | 43.15 (39.70, 45.11) | 40.85 (35.82, 44.45) | **0.0173** |
| TBIL (μmol/L) | | 11.20 (7.47, 18.35) | | 11.25 (7.40, 17.45) | 11.15 (8.48, 31.82) | 0.4010 |
| ALT (U/L) | | 22.00 (16.00, 40.25) | | 22.00 (15.25, 40.00) | 26.50 (16.25, 43.00) | 0.4217 |
| AST (U/L) | | 24.00 (18.75, 42.25) | | 24.00 (18.25, 41.75) | 29.50 (19.25, 43.75) | 0.3483 |
| ALT/AST | | 0.94 (0.73, 1.23) | | 0.94 (0.73, 1.25) | 0.92 (0.73, 1.08) | 0.2517 |
| CA199 (ng/ml) | | 440.71 (51.27, 2584.32) | | 416.23 (53.52, 2526.98) | 687.15 (41.42, 4573.17) | 0.6737 |
| Gender, n (%) | |  | |  |  | 0.0923 |
| Male | | 184 (66.67) | | 157 (64.88) | 27 (79.41) |  |
| Female | | 92 (33.33) | | 85 (35.12) | 7 (20.59) |  |
| Hepatic metastases, n (%) | |  | |  |  | **0.0354** |
| No | | 144 (52.17) | | 132 (54.55) | 12 (35.29) |  |
| Yes | | 132 (47.83) | | 110 (45.45) | 22 (64.71) |  |
| Radical operation, n (%) | |  | |  |  | 0.3244 |
| No | 183 (66.30) | | | 163 (67.36) | 20 (58.82) |  |
| Yes | 93 (33.70) | | | 79 (32.64) | 14 (41.18) |  |

| Continuous values were presented as median (interquartile range) and categorical variables were presented as counts (percentages); Alb: albumin; ALT: alanine aminotransferase; AST: aspartic transaminase; TBIL: total bilirubin. |
| --- |

**QD cohort**

| Variables | Total  (n = 63) | Control  (n = 42) | MASLD  (n = 21) | *P* |
| --- | --- | --- | --- | --- |
|  |  |  |  |  |
| Age (year) | 61.00 (54.00, 69.00) | 61.00 (54.00, 68.25) | 62.00 (60.00, 69.00) | 0.651 |
| Leukocyte (10^9^/L) | 5.83 (4.87, 7.06) | 5.65 (4.70, 7.02) | 6.46 (5.22, 7.92) | 0.107 |
| Neutrophile Granulocyte (10^9^/L) | 3.78 (2.96, 4.86) | 3.47 (2.72, 4.09) | 4.69 (3.61, 5.62) | **0.013** |
| Lymphocyte (mg/L) | 1.30 (1.06, 1.71) | 1.50 (1.09, 1.81) | 1.16 (1.00, 1.39) | 0.139 |
| BUN (mg/dL) | 5.08 (4.10, 5.60) | 5.25 (4.48, 5.83) | 4.45 (3.59, 5.20) | **0.013** |
| UA (μmol/L) | 255.90 (206.20, 309.50) | 266.50 (200.80, 316.80) | 254.00 (224.00, 303.00) | 0.942 |
| Cr (μmol/L) | 75.30 (51.89, 85.85) | 76.10 (52.71, 88.75) | 70.00 (50.09, 82.02) | 0.479 |
| Alb (g/L) | 41.62 (38.48, 43.60) | 41.70 (38.48, 43.55) | 41.44 (38.84, 43.80) | 0.988 |
| GLB (g/L) | 27.80 (25.27, 30.52) | 27.69 (25.23, 30.25) | 27.80 (25.90, 31.35) | 0.382 |
| ALT (U/L) | 24.00 (16.00, 49.77) | 24.90 (16.00, 60.53) | 24.00 (16.00, 34.00) | 0.630 |
| AST (U/L) | 19.00 (15.01, 40.00) | 20.63 (16.00, 40.00) | 17.00 (14.00, 32.00) | 0.311 |
| GGT (U/L) | 40.90 (20.50, 162.00) | 43.66 (20.25, 184.25) | 40.90 (22.60, 153.00) | 0.994 |
| LDH (IU/L) | 171.00 (145.05, 203.00) | 170.30 (150.39, 193.00) | 180.00 (143.00, 244.00) | 0.901 |
| TBIL (μmol /L) | 12.17 (9.36, 20.14) | 11.68 (8.12, 20.27) | 12.38 (10.20, 16.50) | 0.479 |
| DBIL (μmol /L) | 3.97 (3.08, 6.38) | 3.69 (2.84, 6.54) | 4.08 (3.16, 6.04) | 0.462 |
| ALP (IU/L) | 93.00 (71.00, 145.50) | 91.50 (67.00, 153.25) | 103.30 (77.00, 125.51) | 0.867 |
| TG (mmol/L) | 1.22 (0.92, 1.43) | 1.19 (0.99, 1.39) | 1.24 (0.84, 1.51) | 0.385 |
| TC (mmol/L) | 4.95 (4.12, 5.71) | 5.06 (4.15, 5.62) | 4.69 (3.99, 6.04) | 0.983 |
| HDL_C (mmol/L) | 1.19 (1.05, 1.46) | 1.23 (1.11, 1.58) | 1.14 (1.03, 1.24) | 0.091 |
| LDL_C (mmol/L) | 2.85 (2.17, 3.53) | 2.79 (2.06, 3.32) | 3.08 (2.44, 4.20) | 0.258 |
| Lipoprotein (mmol/L) | 233.00 (109.50, 380.39) | 236.00 (110.00, 343.22) | 221.02 (109.00, 448.50) | 0.594 |
| BMI (kg/m^2^) | 22.66 (20.76, 25.69) | 21.62 (20.75, 24.14) | 24.95 (22.66, 25.81) | **0.023** |
| CA199 (U/ml) | 433.70 (87.94, 1000.00) | 585.80 (140.90, 1000.00) | 312.70 (46.81, 989.80) | 0.102 |
| CEA (ng/ml) | 4.88 (3.37, 8.06) | 5.46 (3.70, 7.48) | 4.29 (3.03, 10.66) | 0.924 |
| Gender, n (%) |  |  |  | 0.852 |
| Male | 41 (65.08) | 27 (64.29) | 14 (66.67) |  |
| Female | 22 (34.92) | 15 (35.71) | 7 (33.33) |  |
| Diabetes, n (%) |  |  |  | 0.530 |
| No | 48 (76.19) | 33 (78.57) | 15 (71.43) |  |
| Yes | 15 (23.81) | 9 (21.43) | 6 (28.57) |  |

| Continuous values were presented as median (interquartile range) and categorical variables were presented as counts (percentages); BUN: blood urea nitrogen; UA: uric acid; Cr: creatinine; Alb: albumin; GLB: globulin; ALT: alanine aminotransferase; AST: aspartic transaminase; TBIL: total bilirubin; DBIL: direct bilirubin; LDH: lactic dehydrogenase; GGT: gamma-glutamyl transferase; ALP: alkaline phosphatase; TG: total cholesterol; TC: triglyceride; HDL_C: high-density lipoprotein cholesterol; LDL_C: low-density lipoprotein cholesterol; BMI: body mass index; CEA: carcinoembryonic antigen. |
| --- |

Table S4.

Multivariate logistic regression analysis of PDAC liver metastasis*

| **Variables** | **Total**  **(n = 661)** | **Primary PDAC**  **(n = 464)** | **Liver metastases**  **(n = 197)** | **OR**  **(CI 95％)** | ***P*** |
| --- | --- | --- | --- | --- | --- |
|  |  |  |  |  |  |
| Leukocyte (10^9^/L) | 5.90 (4.87, 7.05) | 5.75 (4.83, 6.90) | 6.10 (5.05, 7.36) | 1.09 (0.85 ~ 1.39) | 0.503 |
| Neutrophile granulocyte (10^9^/L) | 3.76 (2.92, 4.64) | 3.73 (2.86, 4.57) | 4.00 (3.12, 4.96) | 0.89 (0.66 ~ 1.17) | 0.368 |
| ALT (U/L) | 26.00 (15.00, 154.50) | 23.00 (14.00, 136.00) | 30.00 (17.00, 217.00) | 1.00 (1.00 ~ 1.00) | 0.360 |
| LDH (U/L) | 187.00 (162.00, 222.00) | 185.50 (161.75, 215.00) | 189.00 (165.25, 245.50) | 1.00 (1.00 ~ 1.01) | 0.888 |
| CA199 (U/ml) | 175.20 (54.70, 551.85) | 143.95 (49.18, 452.53) | 291.30 (89.15, 796.00) | 1.00 (1.00 ~ 1.00) | 0.217 |
| CEA (ng/ml) | 3.20 (2.04, 5.36) | 3.20 (1.94, 4.90) | 3.52 (2.29, 5.90) | 1.01 (1.00 ~ 1.03) | 0.079 |
| Gender, n (%) |  |  |  |  |  |
| Male | 312 (47.201) | 201 (43.32) | 111 (56.35) | 1.000 (Reference) |  |
| Female | 349 (52.799) | 263 (56.68) | 86 (43.66) | 0.94(0.59 ~ 1.51) | 0.812 |
| Smoke, n (%) |  |  |  |  |  |
| No | 509 (77.00) | 372 (80.17) | 137 (69.54) | 1.00 (Reference) |  |
| Yes | 152 (23.00) | 92 (19.83) | 60 (30.46) | 1.67 (0.99 ~ 2.81) | 0.056 |
| Radical operation, n (%) |  |  |  |  |  |
| No | 244 (36.91) | 134 (28.88) | 110 (55.84) | 1.00 (Reference) |  |
| Yes | 417 (63.09) | 330 (71.12) | 87 (44.16) | 0.27(0.17 ~ 0.41) | <.0001 |
| Comorbidity, n (%) |  |  |  |  |  |
| Ctrl | 357 (54.01) | 292 (62.93) | 65 (32.99) | 1.00 (Reference) |  |
| Diabetes | 134 (20.27) | 108 (23.28) | 26 (13.20) | 1.12 (0.66 ~ 2.14) | 0.562 |
| **MASLD** | **129 (19.52)** | **50 (10.78)** | **79 (40.10)** | 8.10 (4.82 ~ 13.64) | <.0001 |
| **MASLD & Diabetes** | **41(6.20)** | **14 (3.02)** | **27 (13.71)** | 10.40 (4.81~ 22.72) | <.0001 |

*: Factors with P value <0.05 in univariate analysis were selected for multivariate analysis; Continuous values were presented as median (interquartile range) and categorical variables were presented as counts (percentages); ALT: alanine aminotransferase; AST: aspartic transaminase; TBIL: total bilirubin; DBIL: direct bilirubin; LDH: lactic dehydrogenase; GTT: gamma-glutamyltransferase; ALP: alkaline phosphatase; UA: uric acid; BUN: blood urea nitrogen; Cr: creatinine; Alb: albumin; CEA: carcino-embryonic antigen; OR: odds Ratio; CI: confidence Interval.

Table S5.

Multivariate logistic regression analysis of PDAC patients with or without MASLD - BJ cohort (n = 661)

| Variables | Total (n = 661) | Control (n = 491) | MASLD (n = 170) | OR (95%CI) | *P* |
| --- | --- | --- | --- | --- | --- |
| Age (year) | 62.00 (55.00, 68.00) | 63.00 (56.00, 69.00) | 59.50 (53.00, 66.00) | 0.97 (0.95-0.99) | 0.004 |
| Leukocyte (10^9^/L) | 5.88 (4.86, 7.04) | 5.73 (4.81, 6.86) | 6.30 (5.17, 7.53) | 1.21 (0.94 ~ 1.56) | 0.135 |
| Neutrophile Granulocyte (10^9^/L) | 3.76 (2.92, 4.64) | 3.71 (2.86, 4.56) | 4.13 (3.13, 5.08) | 0.93 (0.70 ~ 1.24) | 0.608 |
| ALT (U/L) | 26.00 (15.00, 154.00) | 22.00 (13.00, 122.50) | 38.50 (20.25, 266.00) | 1.00 (1.00 ~ 1.00) | 0.608 |
| AST (U/L) | 23.00 (18.00, 95.00) | 22.00 (17.00, 70.50) | 28.00 (20.00, 139.00) | 1.00 (1.00 ~ 1.01) | 0.886 |
| ALT/AST | 1.05 (0.75, 1.46) | 1.00 (0.72, 1.38) | 1.21 (0.94, 1.69) | 1.15 (0.92 ~ 1.43) | 0.210 |
| TBIL (μmol/L) | 15.00 (10.30, 80.40) | 13.80 (9.70, 44.90) | 19.80 (11.83, 160.48) | 1.02 (1.00 ~ 1.04) | 0.050 |
| DBIL (μmol/L) | 5.20 (3.40, 60.60) | 4.70 (3.20, 32.70) | 7.40 (3.90, 119.60) | 0.98 (0.95 ~ 1.01) | 0.124 |
| GGT (U/L) | 37.00 (19.00, 360.00) | 32.00 (18.00, 291.50) | 76.00 (25.00, 658.25) | 1.00 (1.00 ~ 1.00) | 0.800 |
| LDH (IU/L) | 186.00 (162.00, 219.00) | 184.00 (161.00, 212.50) | 189.50 (163.50, 246.00) | 1.00 (1.00 ~ 1.01) | 0.183 |
| Hepatic metastases, n (%) |  | | | | |
| No | 464 (70.20) | 400 (81.47) | 64 (37.65) | 1.000 (Reference) |  |
| Yes | 197 (29.80) | 91 (18.53) | 106 (62.35) | 7.06 (4.62 ~ 10.78) | <.0001 |

*Factors with *p* value <0.05 in univariate analysis were selected for multivariate analysis; Continuous values were presented as median (interquartile range) and categorical variables were presented as counts (percentages); ALT: alanine aminotransferase; AST: aspartic transaminase; TBIL: total bilirubin; DBIL: direct bilirubin; LDH: lactic dehydrogenase; GTT: gamma-glutamyl transferase; OR: odds ratio; CI: confidence Interval.

Table S6.

Multiple logistic regression of factors associated with PDAC liver metastasis (ZJ cohort, n=276)

| Variables | Unadjusted OR ((95%CI) | *P* | Adjusted OR *(95% CI) | *P* |
| --- | --- | --- | --- | --- |
| Gender |  |  |  |  |
| Male | 1.00 (Reference) |  |  |  |
| Female | 1.00 (0.61 ~ 1.65) | 1.0000 |  |  |
| Radical operation |  |  |  |  |
| No | 1.00 (Reference) |  | 1.00 (Reference) |  |
| Yes | 0.13 (0.07 ~ 0.24) | <.0001 | 0.14 (0.07 ~ 0.28) | <.0001 |
| MASLD |  |  |  |  |
| No | 1.00 (Reference) |  |  |  |
| Yes | 2.20 (1.04 ~ 4.65) | 0.0387 | **3.30** (1.32 ~ 8.24) | 0.0106 |
| Age (year) | 0.98 (0.95 ~ 1.00) | 0.0881 |  |  |
| ALB (g/L) | 1.01 (0.97 ~ 1.05) | 0.8151 |  |  |
| TBIL (μmol/L) | 1.00 (0.99 ~ 1.00) | 0.0401 | 1.00 (0.99 ~ 1.00) | 0.5530 |
| ALT (U/L) | 1.00 (0.99 ~ 1.00) | 0.0088 | 1.00 (0.99 ~ 1.01) | 0.8474 |
| AST (U/L) | 1.00 (0.99 ~ 1.00) | 0.0218 | 1.00 (0.99 ~ 1.01) | 0.5388 |
| ALT/AST | 0.84 (0.53 ~ 1.35) | 0.4778 |  |  |
| CA199 (U/ml) | 1.00 (1.00 ~ 1.00) | 0.0016 | 1.00 (1.00 ~ 1.00) | 0.0371 |

*: Factors with P value <0.05 in univariate analysis were selected for multiple analysis; ALB: albumin; ALT: alanine aminotransferase; AST: aspartic transaminase; TBIL: total bilirubin; OR: odds ratio, CI: confidence interval.

Table S7.

Baseline characteristics of patients with hepatic oligometastatic of PDAC

| **Group** | **Patient-Number** | **Sex** | **Age**  **(year)** | **CA-199**  **(u/ml)** | **BMI**  **(kg/m²)** | **Albumin（g/dl）** | **Globulin（g/dl）** | **ALT**  **(u/l)** | **AST**  **(u/l)** | **Diabetes** | **Smoke** |
| --- | --- | --- | --- | --- | --- | --- | --- | --- | --- | --- | --- |
| Non-MASLD | 1453009 | Female | 52 | 813.8 | 23.15 | 44.5 | 21.6 | 11.8 | 18 | no | yes |
|  | 1783625 | Male | 63 | ＞1000 | 20.76 | 44.6 | 23 | 148.3 | 108.2 | no | yes |
|  | 1509548 | Male | 51 | 7.22 | 17.53 | 35.8 | 24.7 | 188.8 | 101.7 | yes | no |
| MASLD | 1661480 | Male | 54 | ＞1000 | 26.79 | 43.8 | 28.5 | 22.3 | 18.4 | no | no |
|  | 1773267 | Female | 57 | 274.4 | 25.64 | 42.9 | 20.6 | 33.9 | 17.5 | no | no |
|  | 1782116 | Female | 55 | 443.2 | 27.28 | 35.4 | 27.2 | 13.2 | 10.8 | no | no |

Table S8.

Detailed coding of the diagnosis of disease

|  | **Code type** | **Codes** |
| --- | --- | --- |
| MASLD | ICD-9 | 5718 |
|  | ICD-10 | K760, K758 |
| Alcohol-related liver disease | ICD-9 | 5710, 5711, 5712, 5713 |
|  | ICD-10 | K700, K701, K702, K703, K704, K709 |
| PDAC | ICD-9 | 1570, 1571, 1572, 1573, 1578, |
|  | ICD-10 | C250, C251, C252, 253, C257, C258, C259 |
|  | Self-reported | 1026 |
|  | Histology of cancer tumor | 8500 |
| Pancreatic endocrine tumors | ICD-9 | 1574 |
|  | ICD-10 | C254 |
|  | Self-reported | 1026 |
|  | Histology of cancer tumor | 8150, 8240, 8249 |
| Hypertension | ICD-9 | 401, 4010, 4011, 4019 |
|  | ICD-10 | I10, I11, I12, I13, I14, I15 |
| Dyslipidemia | ICD-9 | 27200, 27201, 27202, 27203, 27209, 2721, 2722, 2723, 27240, 27248, 27249, 2725, 2726, 2727, 27280, 27281, 27282, 2729 |
|  | ICD-10 | E780, E781, E782, E783, E784, E785, E786, E788, E789 |
| Diabetes mellitus | ICD-9 | 25000, 25001, 25009, 25010, 25011, 25019, 25020, 25021, 25029, 2502, 2503, 2504, 2505, 2506, 2507, 25090, 25091, 25099 |
|  | ICD-10 | E100, E101, E102, E103, E104, E105, E106, E107, E108, E109, E110, E111, E112, E113, E114, E115, E116, E117, E118, E119, E120, E121, E122, E123, E124, E125, E126, E127, E128, E129, E130, E131, E132, E133, E134, E135, E136, E137, E138, E139, E140, E141, E142, E143, E144, E145, E146, E147, E148, E149 |
| Other tumors | ICD-10 | C00,C01,C02,C03,C04,C05,C06,C07,C08,C09,C10,C11,C12,C13,C14,C15,C16,C17,C18,C19,C20,C21,C22,C23,C24,C26,C30,C31,C32,C33,C34,C37,C38,C39,C40,C41,C43,C44,C45,C46,C47,C48,C49,C50,C51,C52,C53,C54,C55,C56,C57,C58,C60,C61,C62,C63,C64,C65,C66,C67,C68,C69,C70,C71,C72,C73,C74,C75,C76,C77,C78,C79C,C80,C81,C82,C83,C84,C85,C86,C88,C90,C91,C92,C93,C94,C95,C96,C97,D00,D01,D02,D03,D04,D05,D06,D07,D09 |
|  | Self-reported | 20001 |

Table S9.

Frequencies and percentages of missing data according to population enrolled

|  | **Total** | **Not diagnosed with MASLD** | **MASLD** |
| --- | --- | --- | --- |
| Number | **450,754** | **444,025** | **6,729** |
| Waist circumference, *n* (%) | 78 (0.017) | 76 (0.017) | 2 (0.030) |
| Hip circumference, *n* (%) | 70 (0.015) | 70 (0.015) | / |
| Diastolic pressure, *n* (%) | 372 (0.083) | 356 (0.080) | 16 (0.238) |
| Systolic pressure, *n* (%) | 384 (0.085) | 368 (0.082) | 16 (0.238) |
| Townsend deprivation index, *n* (%) | 11,375 (2.524) | 11,192 (2.520) | 183 (2.720) |
| AST, *n* (%) | 29,634 (6.574) | 29,092 (6.551) | 542 (8.055) |
| ALT, *n* (%) | 28191 (6.254) | 27,664 (6.230) | 527 (7.832) |
| Cholesterol, *n* (%) | 28029 (6.218) | 27510 (6.196) | 519 (7.713) |
| LDL-C, *n* (%) | 28,823 (6.394) | 28,282 (6.369) | 541 (8.040) |
| GGT, *n* (%) | 28,210 (6.258) | 27,686 (6.235) | 524 (7.787) |
| Triglycerides, *n* (%) | 28,357 (6.291) | 27,824 (6.266) | 533 (7.921) |
| UA, *n* (%) | 28,534 (6.330) | 28,008 (6.308) | 526 (7.817) |

Table S10.

Field codes for Blood biochemical indicators and illness history of interest in the UK Biobank

| **Biomarkers and illness** | **Field codes** |
| --- | --- |
| Low-density lipoprotein cholesterol | 30780 |
| Triglycerides | 30870 |
| Cholesterol | 30690 |
| Gamma glutamyl transferase | 30730 |
| Urate | 30880 |
| Aspartate aminotransferase | 30650 |
| Alanine aminotransferase | 30620 |
| hypertension | 1065 |
|  | 1072 |
| Diabetes | 1220 |
|  | 1222 |
|  | 1223 |
|  | 1276 |
|  | 1468 |
|  | 1607 |
| Dyslipidemia | 1473 |

Table S11.

The Akaike information criterion and the Bayesian information criterion in model selection

|  | **Model 1** | **Model 2** | **Model 3** |
| --- | --- | --- | --- |
| MASLD |  |  |  |
| AIC | 71,942.8 | 71,812.4 | 71,780.2 |
| BIC | 71,996.6 | 71,865.8 | 71,851.4 |

Abbreviations: AIC, Akaike information criterion; BIC, Bayesian information criterion; MASLD, Metabolic Dysfunction Associated Steatotic Liver Disease

Table S12.

Associations between severe NAFLD and PC after excluding individuals diagnosed with PC within less than 1 year of exposure to severe MASLD

|  | **Hazard ratio (95% CI)** | **P value** |
| --- | --- | --- |
|  | **MASLD exposure (time varying)** |  |
| MASLD |  |  |
| Model 1^a^ | 2.00 (1.37, 2.90) | 0.0003 |
| Model 2^b^ | 1.74 (1.20, 2.53) | 0.0037 |
| Model 3^c^ | 1.62 (1.11, 2.35) | 0.0123 |
| CI, confidence interval. ^a^ Model 1, adjusted for sex, age, and Indices of Multiple Deprivation, ^b^ Model 2, included covariates from model 1 plus drinking status, smoking status, body mass index. ^c^ Model 3, included covariates from model 2 plus TG, history of hypertension and history of diabetes, TG: triglycerides. | | |

Table S13.

Associations between severe MASLD and PC after adding more variables

|  | **Hazard ratio (95% CI)** | **P value** |
| --- | --- | --- |
|  | **MASLD exposure (time varying)** |  |
| MASLD |  |  |
| Model 1^d^ | 3.48 (2.69, 4.50) | <0.0001 |
| Model 2^e^ | 3.44 (2.66, 4.46) | <0.0001 |
| Model 3^f^ | 3.33 (2.56, 4.32) | <0.0001 |
| CI, confidence interval. ^d^ Model 1, adjusted for sex, age, and Indices of Multiple Deprivation, drinking status, smoking status, body mass index, TG, history of hypertension, history of diabetes and ethnicity, ^e^ Model 2, included covariates from model 1 plus central obesity and history of dyslipidemia. ^f^ Model 3, included covariates from model 2 plus AST, ALT, GGT and LDL-C, TG: triglycerides; AST: aspartic transaminase; ALT: alanine aminotransferase; GGT: gamma-glutamyl transferase; LDL_ C: low density lipoprotein | | |

Table S14.

Association between severe MASLD and PC after excluding severe MASLD at baseline

|  | **Hazard ratio (95% CI)** | **P value** |
| --- | --- | --- |
|  | **MASLD exposure (time varying)** |  |
| MASLD |  |  |
| Model 1^a^ | 5.42 (4.20, 7.01) | < 0.0001 |
| Model 2^b^ | 4.72 (3.64, 6.10) | < 0.0001 |
| Model 3^c^ | 4.48 (3.46, 5.80) | < 0.0001 |
| CI, confidence interval. ^a^ Model 1, adjusted for sex, age, and Indices of Multiple Deprivation, ^b^ Model 2, included covariates from model 1 plus drinking status, smoking status, body mass index. ^c^ Model 3, included covariates from model 2 plus TG, history of hypertension and history of diabetes, TG: triglycerides. | | |

Table S15.

Associations between severe MASLD and PC after excluding missing data

|  | **Hazard ratio (95% CI)** | **P value** |
| --- | --- | --- |
|  | **MASLD exposure (time varying)** |  |
| MASLD |  |  |
| Model 1^a^ | 4.38 (3.35, 5.73) | < 0.0001 |
| Model 2^b^ | 3.84 (2.93, 5.03) | < 0.0001 |
| Model 3^c^ | 2.86 (2.18, 3.75) | < 0.0001 |
| CI, confidence interval. ^a^ Model 1, adjusted for sex, age, and Indices of Multiple Deprivation, ^b^ Model 2, included covariates from model 1 plus drinking status, smoking status, body mass index. ^c^ Model 3, included covariates from model 2 plus TG, history of hypertension and history of diabetes, TG: triglycerides. | | |

Data S1. (separate file)

All antibodies were listed in Data S1.

Data S2. (separate file)

The mIHC staining panels were listed in Data S2.

Data S3. (separate file)

KEGG analysis of upregulated pathways in NAFLD group (n=3 per group).

Data S4. (separate file)

GSEA analysis listing all gene sets enriched in phenotype NAFLD group (n=3 per group).

Data S5. (separate file)

Gene sets associated with pluripotency in NAFLD group (n=3/group).

Data S6. (separate file)

IPG1576 profile summary.

**References**

1 Kim, H. N. *et al.* CT-based Hounsfield unit values reflect the degree of steatohepatitis in patients with low-grade fatty liver disease. *BMC Gastroenterol* **23**, 77 (2023).

2 Sudlow, C. *et al.* UK biobank: an open access resource for identifying the causes of a wide range of complex diseases of middle and old age. *PLoS Med* **12**, e1001779 (2015).

3 Chen, L., Fan, Z. & Lv, G. Associations of muscle mass and grip strength with severe NAFLD: A prospective study of 333,295 UK Biobank participants. *J Hepatol* **77**, 1453-1454 (2022).

4 Hagström, H. *et al.* Administrative Coding in Electronic Health Care Record-Based Research of NAFLD: An Expert Panel Consensus Statement. *Hepatology* **74**, 474-482 (2021).

5 Chen, C. *et al.* Associations of severe liver diseases with cataract using data from UK Biobank: a prospective cohort study. *EClinicalMedicine* **68**, 102424 (2024).

6 Liu, M. *et al.* Accelerometer-derived moderate-to-vigorous physical activity and incident nonalcoholic fatty liver disease. *BMC Med* **22**, 398 (2024).

7 Yang, H. *et al.* Physical frailty, genetic predisposition, and the risks of severe non-alcoholic fatty liver disease and cirrhosis: a cohort study. *J Cachexia Sarcopenia Muscle* **15**, 1491-1500 (2024).

8 Xiao, L. *et al.* Polygenic risk score of metabolic dysfunction-associated steatotic liver disease amplifies the health impact on severe liver disease and metabolism-related outcomes. *J Transl Med* **22**, 650 (2024).

9 Hayati Rezvan, P., Lee, K. J. & Simpson, J. A. The rise of multiple imputation: a review of the reporting and implementation of the method in medical research. *BMC Med Res Methodol* **15**, 30 (2015).

10 Lévesque, L. E., Hanley, J. A., Kezouh, A. & Suissa, S. Problem of immortal time bias in cohort studies: example using statins for preventing progression of diabetes. *Bmj* **340**, b5087 (2010).

11 Hwang, P. H. *et al.* Ophthalmic conditions associated with dementia risk: The Cardiovascular Health Study. *Alzheimers Dement* **17**, 1442-1451 (2021).

12 Lee, C. S. *et al.* Association Between Cataract Extraction and Development of Dementia. *JAMA Intern Med* **182**, 134-141 (2022).

13 Park, D. Y., Kim, M., Bae, Y., Jang, H. & Lim, D. H. Risk of Dementia in Newly Diagnosed Glaucoma: A Nationwide Cohort Study in Korea. *Ophthalmology* **130**, 684-691 (2023).

14 Hao, Y. *et al.* Integrated analysis of multimodal single-cell data. *Cell* **184**, 3573-3587.e3529 (2021).

15 Jin, S. *et al.* Inference and analysis of cell-cell communication using CellChat. *Nat Commun* **12**, 1088 (2021).

16 Yachida, S. *et al.* Distant metastasis occurs late during the genetic evolution of pancreatic cancer. *Nature* **467**, 1114-1117 (2010).

17 Wang, L., Xu, R., Kaelber, D. C. & Berger, N. A. Glucagon-Like Peptide 1 Receptor Agonists and 13 Obesity-Associated Cancers in Patients With Type 2 Diabetes. *JAMA Netw Open* **7**, e2421305 (2024).
